# Deep exome sequencing identifies enrichment of deleterious mosaic variants in neurodevelopmental disorder genes and mitochondrial tRNA regions in bipolar disorder

**DOI:** 10.1038/s41380-023-02096-x

**Published:** 2023-05-30

**Authors:** Masaki Nishioka, Jun Takayama, Naomi Sakai, An-a Kazuno, Mizuho Ishiwata, Junko Ueda, Takashi Hayama, Kumiko Fujii, Toshiyuki Someya, Shinichi Kuriyama, Gen Tamiya, Atsushi Takata, Tadafumi Kato

**Affiliations:** 1 Department of Psychiatry and Behavioral Science, Juntendo University Graduate School of Medicine, 2-1-1 Hongo, Bunkyo-Ku, Tokyo, 113-8421 Japan; 2 Department of Molecular Pathology of Mood Disorders, Juntendo University Graduate School of Medicine, 2-1-1 Hongo, Bunkyo-Ku, Tokyo, 113-8421 Japan; 3 Laboratory for Molecular Dynamics of Mental Disorders, RIKEN Center for Brain Science, 2-1 Hirosawa, Wako, Saitama 351-0198 Japan; 4 Laboratory for Molecular Pathology of Psychiatric Disorders, RIKEN Center for Brain Science, 2-1 Hirosawa, Wako, Saitama 351-0198 Japan; 5 Department of AI and Innovative Medicine, Tohoku University School of Medicine, 2-1 Seiryo-machi, Aoba-Ku, Sendai, Miyagi 980-8575 Japan; 6 Department of Integrative Genomics, Tohoku Medical Megabank Organization, Tohoku University, 2-1 Seiryo-machi, Aoba-Ku, Sendai, Miyagi 980-8573 Japan; 7 Statistical Genetics Team, RIKEN Center for Advanced Intelligence Project, 1-4-1 Nihonbashi, Chuo-ku, Tokyo, 103-0027 Japan; 8 Yokohama Mental Clinic Totsuka, 494-8 Kamikurata-cho, Totsuka-ku, Yokohama, 244-0816 Japan; 9 Department of Psychiatry, Shiga University of Medical Science, Seta Tsukinowa-Cho, Otsu, Shiga 520-2192 Japan; 10 Department of Psychiatry, Niigata University Graduate School of Medical and Dental Sciences, 757 Asahimachidori-ichibancho, Chuo-ku, Niigata, 951-8510 Japan; 11 Department of Preventive Medicine and Epidemiology, Tohoku Medical Megabank Organization, Tohoku University, 2-1 Seiryo-machi, Aoba-Ku, Sendai, Miyagi 980-8573 Japan; 12 Department of Molecular Epidemiology, Tohoku University School of Medicine, 2-1 Seiryo-machi, Aoba-Ku, Sendai, Miyagi 980-8575 Japan; 13 Research Institute for Diseases of Old Age, Juntendo University Graduate School of Medicine, 2-1-1 Hongo, Bunkyo-Ku, Tokyo, 113-8421 Japan

**Keywords:** Bipolar disorder, Genetics

## Abstract

Bipolar disorder (BD) is a global medical issue, afflicting around 1% of the population with manic and depressive episodes. Despite various genetic studies, the genetic architecture and pathogenesis of BD have not been fully resolved. Besides germline variants, postzygotic mosaic variants are proposed as new candidate mechanisms contributing to BD. Here, we performed extensive deep exome sequencing (DES, ~300×) and validation experiments to investigate the roles of mosaic variants in BD with 235 BD cases (194 probands of trios and 41 single cases) and 39 controls. We found an enrichment of developmental disorder (DD) genes in the genes hit by deleterious mosaic variants in BD (*P* = 0.000552), including a ClinVar-registered pathogenic variant in *ARID2*. An enrichment of deleterious mosaic variants was also observed for autism spectrum disorder (ASD) genes (*P* = 0.000428). The proteins coded by the DD/ASD genes with non-synonymous mosaic variants in BD form more protein-protein interaction than expected, suggesting molecular mechanisms shared with DD/ASD but restricted to a subset of cells in BD. We also found significant enrichment of mitochondrial heteroplasmic variants, another class of mosaic variants, in mitochondrial tRNA genes in BD (*P* = 0.0102). Among them, recurrent m.3243 A > G variants known as causal for mitochondrial diseases were found in two unrelated BD probands with allele fractions of 5–12%, lower than in mitochondrial diseases. Despite the limitation of using peripheral tissues, our DES investigation supports the possible contribution of deleterious mosaic variants in the nuclear genome responsible for severer phenotypes, such as DD/ASD, to the risk of BD and further demonstrates that the same paradigm can be applied to the mitochondrial genome. These results, as well as the enrichment of heteroplasmic mitochondrial tRNA variants in BD, add a new piece to the understanding of the genetic architecture of BD and provide general insights into the pathological roles of mosaic variants in human diseases.

## Introduction

Bipolar disorder (BD) afflicts around 1% of the population with depressive and manic episodes. The sufferings of the patients and the societal cost [1] necessitate the development of effective therapeutic management. Although current medications are indispensable for patients, they are not perfect solutions due to potential adverse effects and treatment resistance in many cases. We need to understand the biological mechanisms of BD to develop better treatment for future psychiatry [2].

Encouraged by the high heritability of BD [3], various genomic approaches have started to elucidate the biological mechanisms of BD. Large-scale genome-wide association studies revealed many associated loci [4–6]. Whole-exome sequencing (WES) with multiplex families suggested potentially disease-associated genes [7, 8]. Trio-based WES studies found the relevance of the high probability of being loss-of-function intolerant (pLI) genes [9–11]. A large-scale WES study reported one promising candidate gene, *AKAP11* [12]. Short copy number variations in *ASTN2, DLG2*, and *PCDH15* were associated with BD [13]. While these studies have contributed to our understanding of BD, the associated loci and genes are not enough to fully explain the biological mechanisms of BD.

Besides germline variants, postzygotic mosaic variants arising during development are new candidate mechanisms explaining a remaining part of the genetic architecture of psychiatric disorders [14, 15]. Mosaic variants have been indicated as components of the genetic architecture of autism spectrum disorder (ASD) [16–21] and schizophrenia [22–24]. Inspired by our preliminary data of mosaic variants in BD, we proposed a hypothesis that deleterious mosaic de novo variants (mDNVs) in the genes associated with developmental disorder (DD) by germline de novo variants (gDNVs) would contribute to BD [9]. However, our previous analysis using conventional WES data (~50× coverage) had an inherent limitation in the sensitivity of mosaic variants. The mosaic variants remain to be extensively investigated to decipher the enigmatic genetic architecture of BD.

**Fig. 1:**
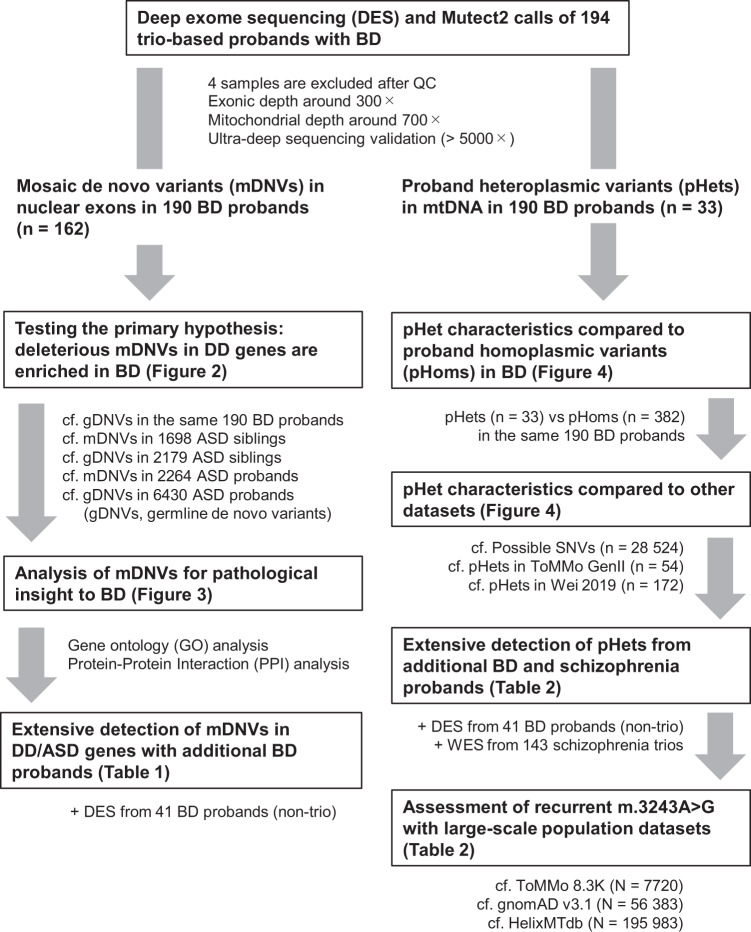
An overview of our study design. A schematic illustration of the workflow of our study. BD bipolar disorder, DD developmental disorder, WES conventional (non-deep) whole-exome sequencing.

Here, we tested our proposed hypothesis by trio-based deep exome sequencing (DES, around 300×) with an expanded cohort of BD (*N* = 235: 194 trio-based probands and 41 non-trio cases) and 39 controls. We used peripheral tissues for DES to detect mosaic variants of early developmental origin that should be basically shared between the brain and peripheral tissues. Bae et al. reported that over 90% of the mosaic variants with variant allele fractions (VAFs) above 2% in at least one brain region are detectable in another tissue [25], which means peripheral tissues are useful surrogates when targeting potentially relevant mosaic variants in the brain. Indeed, several studies have reported the contribution of mosaic variants to ASD using peripheral tissues [18–21]. We also sought another class of mosaic variants, mitochondrial heteroplasmic variants, to extend our initial hypothesis of mosaic variants (study design in Fig. 1). We found the enrichment of deleterious mDNVs in DD and ASD genes, supporting the association of mosaic variants in DD/ASD genes and BD. We also found the enrichment of heteroplasmic variants in mitochondrial tRNA genes in BD, including recurrent m.3243 A > G variants, one of the most major causal variants of mitochondrial diseases (a subtype of DD), indicating mitochondrial tRNA variants as a new candidate background of BD.

## Materials and methods

See Supplementary Methods for details.

### Study participants

We recruited 194 BD probands with their parents (trios), 41 BD single cases, and age-matched 39 healthy controls without psychiatric disorders, including 18 unaffected siblings of BD probands. The BD participants were clinically diagnosed with BD or schizoaffective disorder (SCZAD) by trained psychiatrists, verified by the Diagnostic and Statistical Manual of Mental Disorders as previously [10]. The saliva or blood was collected after obtaining written informed consent. Among the 235 BD participants, 131 overlapped with our previous study [9], and 104 were newly recruited. This study was designed according to the Helsinki declaration and approved by the Research Ethics Committee, Faculty of Medicine, Juntendo University, RIKEN Wako Research Ethics First Committee, the Ethics Committee on Genetics of Niigata University, and the Research Ethical Committee of Tohoku Medical Megabank Organization, Tohoku University.

### Deep exome sequencing

The DNA from the BD (*N* = 235) and control (*N* = 39) participants newly underwent DES. The exonic and mitochondrial DNA was enriched by SureSelect Human All Exon v5/6 + mitochondrial probes (Agilent Technologies, Santa Clara, CA, USA) and sequenced by NovaSeq6000 or NextSeq2000 (Illumina, San Diego, CA, USA) at a theoretical depth of 500×. The sample data are summarized in Table S1. A BD sample with low depth (<100×) was omitted (*N* = 1).

### Read alignment and variant calling

We performed an alignment and quality control based on the GATK Best Practice [26–28] using GATK-4.0/4.1 with two reference genomes (hg38 and hs37d5) (Table S2). We called the candidate exonic and mitochondrial mosaic variants in trio-based BD probands by Mutect2 [29] with parental sequencing data to filter out transmitted variants and sequencing artifacts. The non-trio-based BD cases (*N* = 41) and controls (*N* = 39) underwent Mutect2 calling without parental data. We used our in-house Panel of Normals (PoN) (Supplementary Data), the PoN by Broad Institute, and gnomAD-r2.1.1 non-PASS alleles [30] to exclude sequencing artifacts. We selected the final exonic mDNVs and the mitochondrial heteroplasmic variants in the probands (pHets) by hard filtering (Table S2). The 41 non-trio-based BD cases and 39 controls were limited to exploratory analysis. For trio-based BD probands, the outliers in per-individual mDNV counts that deviated from the expected Poisson distributions were excluded from the subsequent analysis (*N* = 3), resulting in 190 trios for the subsequent analysis.

The gDNVs in the same trio-based BD probands (*N* = 190) were partly compiled from our previous study [9] and additionally called for new trios, using GATK-HaplotypeCaller, triodenovo-0.06 [31], and DNMFilter-0.1.1 [32] (Table S2). The mitochondrial homoplasmic variants in the BD probands (pHoms) were called by GATK-HaplotypeCaller. Since the analytical engine of HaplotypeCaller is shared with Mutect2, the calling bias should be consistent between the mDNVs and gDNVs or between pHets and pHom. The variants detected by the hs37d5 pipeline underwent liftover to the hg38 coordinate for variant annotations.

### Variant annotation

The exonic variants were annotated with gnomAD-r2.1.1 [30], ToMMo-8.3KJPN (Japanese population dataset) [33], SnpEff-4.3 [34], MPC [35], and dbNSFP-4.0a [36] (seven algorithms for missense variants: SIFT, PolyPhen-2 HumVar/HumDiv, LRT, MutationTaster, MutationAssessor, and PROVEAN [37–42]). We defined deleterious variants as loss-of-function (LoF: nonsense, frameshift, and canonical splice) or damaging missense (MPC ≥ 2 or predicted as damaging by all the seven algorithms above). The mitochondrial variants were annotated with gnomAD-v3.1 [43], ToMMo-8.3KJPN [33], HelixMTdb [44], SnpEff-4.3 [34], MitoTIP [45], and PON-mt-tRNA [46].

### Validation of mosaic variants

The candidate mosaic variants underwent target amplicon sequencing (TAS) validation by MiSeq/iSeq100 (Illumina) (median 26 719×). For pHets, the candidates underwent TAS for all the trio members to exclude the possibility of misalignment due to nuclear mitochondrial DNA (NUMT) [47]. TAS libraries were prepared and analyzed as previously [9, 48, 49]. The variants failing in TAS validation were excluded from the subsequent analysis.

### The enrichment analysis of mDNVs

We assessed the enrichment of DD and ASD genes in the genes hit by mDNVs and gDNVs in BD, ASD probands, and unaffected ASD siblings by DNENRICH [50] with one million permutations. The gDNVs/mDNVs in ASD probands and unaffected ASD siblings were compiled from previous ASD studies [18, 51] as positive and negative controls, respectively. The DD genes (285 genes) were defined as genes associated with DD through gDNVs in Kaplanis et al. [52]. The ASD genes (924 genes) were defined as SFARI scores 1–3. We limited the comparison to single nucleotide variants (SNVs) because insertion/deletions (INDELs) are sensitive to the pipeline difference.

We performed gene ontology (GO) analysis by DNENRICH [50] with one million permutations using the same gene-set and procedure as previously [9]. We used STRING [53] for protein-protein interaction (PPI) analysis (https://string-db.org/) and Cytoscape-v3.7.2 [54] for visualization.

We analyzed DES data from the age-matched 39 controls of our recruitment, including 18 unaffected siblings of BD probands, to rule out apparent technical artifacts and assess the possibility of mosaic variants due to clonal hematopoietic expansion frequently observed in individuals without psychiatric disorders.

### Heteroplasmic variant analysis

We classified the mitochondrial genic variants into four classes: synonymous, non-synonymous, tRNA, and rRNA. The intergenic regions were excluded because of much higher mutation rates than the genic regions [55]. We limited the analysis to SNVs, excluding low-mappability regions (Supplementary Data). First, we compared the proportions of target variants to total genic variants in the pHets and other variant sets: pHoms of the same BD probands (*N* = 190) and all the possible mitochondrial genic variants. We also checked the characteristics of pHets in two population datasets as references: ToMMo trio generation II (ToMMo GenII, *N* = 518 trios) [33, 56, 57] and Wei et al. [55] (*N* = 1526 duos). We called the pHets in ToMMo dataset using the matching procedure for our BD. We compiled the pHets in Wei et al. [55], which extensively studied mitochondrial heteroplasmic variants in mother-proband duos, selecting de novo heteroplasmic variants in their original publication. The schizophrenia trio data (*N* = 615 trios) were derived from Fromer et al. [50] (dbGaP phs000687.v1.p1) with authorization and processed by the matching procedure for the BD trios. The statistical assessments for rare proportions were performed by Fisher’s exact test (FET).

## Results

### The enrichment of the DD/ASD genes in mDNVs in BD

We obtained DES data at ~300× non-duplicated depth for exonic regions (median depth = 296× [hg38] and 332× [hs37d5] for BD cases and 300× [hg38] and 337× [hs37d5] for controls, Table S3). The depth in hg38 was lower than in hs37d5 due to the alignment to alternative contigs. The estimated contamination rates were low (<0.2%) enough for detecting mosaic variants with low allelic fractions. We detected 162 high-confidence exonic mDNVs from 190 trio-based BD probands (Table S4), including 27 mDNVs detected previously [9]. In the 131 probands commonly analyzed in our current and previous studies [9], we identified 119 mDNVs, whereas only 27 of them were detected in our previous conventional WES study [9]. This 4.4-fold increase clearly indicates the advantage of DES. The sequencing data of the parents as technical controls facilitated the effective removal of sequencing artifacts, achieving a validation rate of 93.8% for exonic mDNVs (SNV 94.3% [116/123]; INDEL 80% [4/5]). The detection sensitivity was satisfactory (162/190 = 0.85 exonic mDNV per sample), consistent with the theoretical estimate of one exonic mDNV per sample with an even sequencing depth of 300× [14]. The similarity of the mutational patterns of mDNVs in BD and gDNVs in unaffected ASD siblings [51] enabled us to apply the theoretical mutation rates in DNENRICH, originally designed for gDNVs, to mDNVs (cosine similarity = 0.901, Fig. S1).

**Fig. 2:**
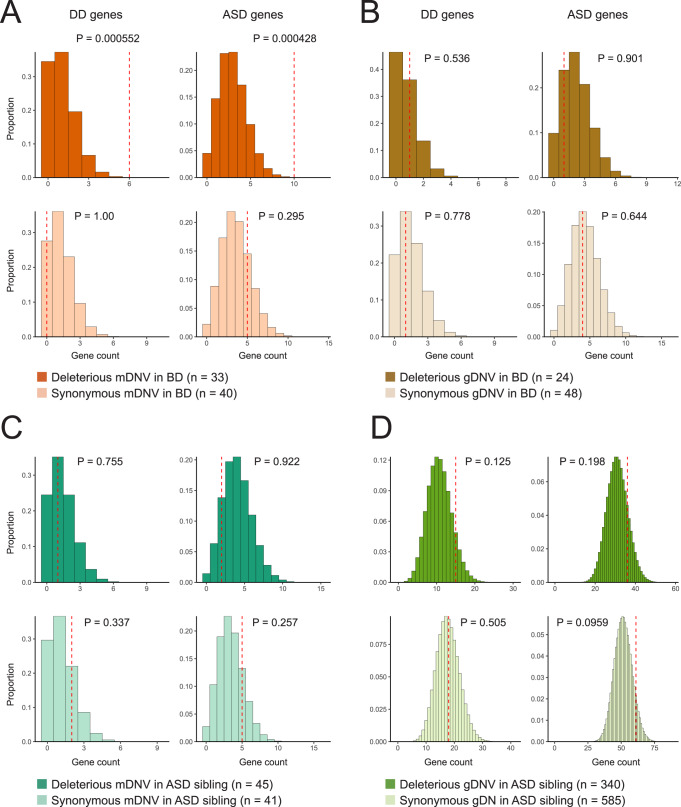
The enrichment of DD/ASD genes in the genes hit by mDNVs in BD. The histogram illustrates the expected number of mDNVs/gDNVs hitting the two gene sets (DD and ASD genes, the x-axis) and their relative frequency (the y-axis). DNENRCIH calculated the distribution with one million random permutations. The red dotted line indicates the observed number of variants in DD/ASD genes. The probability of the observed number or more in the simulated distribution is described near the red dotted line. **A** Deleterious and synonymous mDNVs in BD. **B** Deleterious and synonymous gDNVs in BD. **C** Deleterious and synonymous mDNVs in unaffected ASD siblings. **D** Deleterious and synonymous gDNVs in unaffected ASD siblings.

First, we tested our primary hypothesis of the possible enrichment of DD genes in the genes hit by deleterious mDNVs with theoretical expectations using DNENRICH, which considers gene length and trinucleotide mutational contexts. While the genes hit by synonymous mDNVs in BD were not enriched in DD genes (*P* = 1.00), the genes hit by deleterious mDNVs in BD were enriched in DD genes (*P* = 0.000552) (Fig. 2A). We also observed the enrichment of ASD genes in the genes hit by deleterious mDNVs in BD (*P* = 0.000428) but not in the genes hit by synonymous mDNVs in BD (*P* = 0.295) (Fig. 2A). While the data came from analysis with peripheral tissues, these results extend the categories of genes in the primary hypothesis from DD genes to DD/ASD genes. In contrast, these enrichments were not observed for the deleterious gDNVs in the same BD probands (Table S5, Fig. 2B) and the mDNVs/gDNVs in unaffected ASD siblings compiled from previous ASD studies [18, 51] (Fig. 2C, D). No enrichment of DD/ASD genes in the genes hit by mDNVs in the unaffected ASD siblings as negative control individuals indicates that the DD/ASD genes are not naturally vulnerable to mosaic mutations more than theoretical expectations. Reassuringly, the same analytical procedure confirmed the enrichments of DD/ASD genes in the gene hit by deleterious mDNVs/gDNVs in ASD probands as positive controls [18, 51] (Fig. S2). These contrasts still held when limiting the mDNVs to LoF variants (Fig. S3). The enrichment of DD/ASD genes in the genes hit by deleterious mDNVs in BD was significant even after stringent Bonferroni correction (corrected *P* = 0.0199 and 0.0154 for DD and ASD genes, respectively, *n* = 36 tests). Based on the fold change of deleterious mDNVs in DD genes in BD to the theoretical expectation (5.73-fold, 6/190 samples), the statistical power from the sample size of 190 was 0.89 at the α-level of 0.05, supporting our observation.

### DD genes of interest hit by deleterious mDNVs

Subsequently, we took a closer look at the individual deleterious (LoF and damaging missense) mDNVs in DD/ASD genes (Table 1). Of note, one BD proband had an LoF mDNV in *ARID2* (p.Arg1769*), a recurrent variant observed in multiple patients with Coffin-Siris syndrome as germline variants (ClinVar Accession: VCV000451913.4). Coffin-Siris syndrome is a severe DD characterized by intellectual disability, delayed development of speech and motor skills, facial features, and hypoplasia of fingers or toes. This fact supported the potential pathogenicity of the LoF mDNV in *ARID2* for BD. The pathogenic relevance of an LoF mDNV in *KMT2C*, another robust causal mutation for DD if present as gDNVs, was discussed previously [9].

**Table 1:** The deleterious mDNVs in DD/ASD genes in BD.

Family ID	Diagnosis	Gene	Variant^a^	Type	HGVS.p	VAF (%)	DD by gDNVs	SFARI score	gnomAD non_neuro.AF	ToMMo AF
**Trio-based**										
204	BDI	*AGO2*	chr8:140539427 G > A	Damaging missense	p.Arg688Cys	3.0	.	2	0	0
215	BDI	*KMT2C*	chr7:152162776 T > A^#^	Nonsense	p.Lys3601*	26.8	Kleefstra syndrome	1	0	0
216	BDI	*CACNB1*	chr17:39187576 C > G	Damaging missense	p.Arg106Pro	1.3	.	3	0	0
223	BDII	*PLXNB1*	chr3:48412912 C > T^#^	Damaging missense	p.Gly1562Ser	8.5	.	2	0	0
301	BDII	*ARID2*	chr12:45893663 C > T	Nonsense	p.Arg1769*	5.4	Coffin-Siris syndrome	3	0	0
306	BDI	*SRCAP*	chr16:30712771 C > T^#^	Damaging missense	p.Leu696Phe	4.3	Floating-Harbor syndrome	1	0	0
312	BDII	*HIVEP2*	chr6:142764814 C > T^#^	Damaging missense	p.Ala1835Thr	18.5	Intellectual disability	1	0	0
314	BDI	*SLC35A2*	chrX:48909915 C > T	Damaging missense	p.Arg86His	2.3	Congenital disorder of glycosylation	.	0	0
431	BDI	*UNC13A*	chr19:17645808 C > T	Damaging missense	p.Arg741His	2.5	.	3	0	0
431	BDI	*GNAS*	chr20:58853587 C > T	Nonsense	p.Gln108*	2.4	McCune-Albright syndrome	3	0	0
475	BDI	*SSPO*	chr7:149777848 C > T	Nonsense	p.Gln246*	2.3	.	3	0	0
509	BDI	*ARHGAP32*	chr11:128970238 G > A	Damaging missense	p.Arg1645Trp	1.9	.	3	0	0
**Non-trio-based**										
540	BDI	*BCL11B*	chr14:99176061 G > A	Damaging missense	p.Leu259Phe	2.8	Intellectual developmental disorder	.	0	0
572	BDI	*MED13*	chr17:61984807 CAT > CAATGAATATGAAAATCCCAATG	Frameshift	p.Met845Hisfs*13	1.4	.	1	0	0
573	BDI	*NACC1*	chr19:13135277 C > T	Damaging missense	p.Arg24Trp	31.7	Neurodevelopmental disorder with epilepsy	1	0	0
574	BDI	*CHAMP1*	chr13:114324952 G > A	Nonsense	p.Trp370*	11.6	Intellectual disability	1	0	0

*BDI* Bipolar I disorder, *BDII* Bipolar II disorder.

^a^# indicates mDNVs previously detected in our conventional WES study [9].

**Fig. 3:**
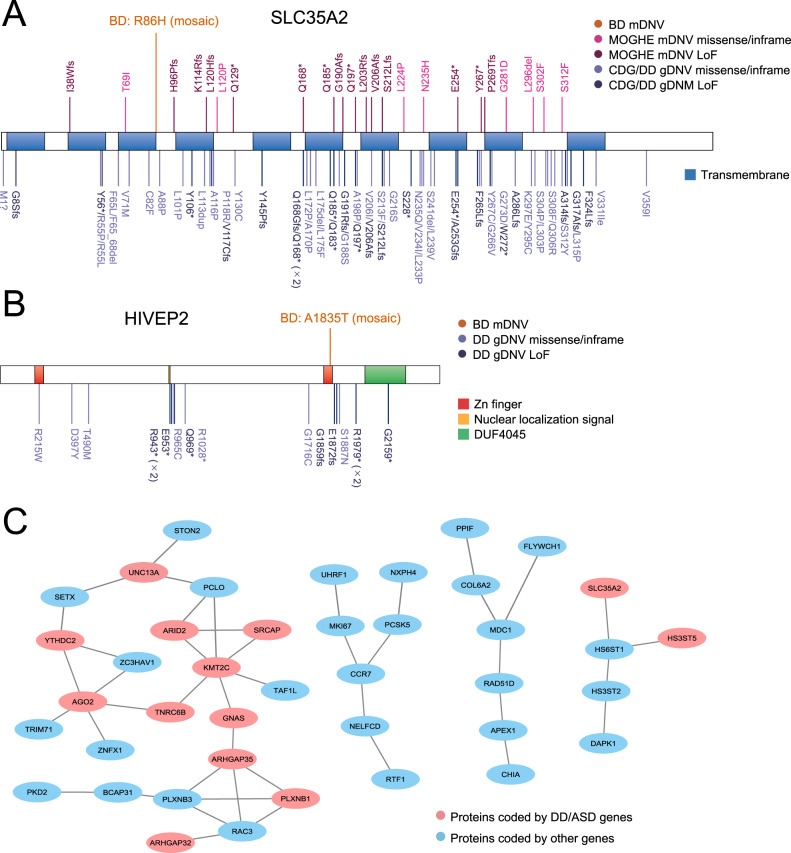
Mechanistic insight into the DD/ASD genes hit by non-synonymous mDNVs. **A** The positions of mDNVs/gDNVs on the SLC35A2 protein in BD and other diseases. The mDNVs in MOGHE (malformation of cortical development with oligodendroglial hyperplasia in epilepsy) [58] are indicated on the upper side; the gDNVs in DD [52] (including CDG [congenital disorders of glycosylation] [59]) are on the lower side. **B** The positions of mDNVs/gDNVs on the HIVEP2 protein in BD and other diseases. The mDNVs in BD hit the Zn finger domain. The gDNVs in DD [52] are indicated on the lower side. **C** The PPI network of the genes hit by non-synonymous mDNVs in BD. The red circles indicate the proteins coded by DD/ASD genes, and the blue circles indicate others.

Among damaging missense mDNVs, the missense mDNV in *SLC35A2* was of particular interest because several mDNVs in *SLC35A2* are causal for malformation of cortical development with oligodendroglial hyperplasia in epilepsy (MOGHE), an epileptic syndrome with neuropathological abnormality in the brain [58], while gDNVs in *SLC35A2* are causal for a developmental disorder, congenital disorders of glycosylation (CDG) [59] (Fig. 3A). The mDNV in *SLC35A2* in BD would also have a pathological effect in specific brain regions. The damaging missense mDNV in *HIVEP2*, a gene coding a brain-expressed transcription factor, hit a conserved amino acid in the DNA binding domain (Fig. 3B), the essential region of the DNA-binding transcription factor. The damaging missense mDNV in *SRCAP*, which hit the DNA-binding domain of SRCAP protein consisting of a chromatin-remodeling complex, was previously analyzed in detail [9]. Overall, the damaging missense mDNVs in the DD/ASD genes were positioned at the genomic regions coding well-conserved amino acids (Fig. S4), supporting their potential pathogenicity. As expected, none of the 12 deleterious mDNVs in DD/ASD genes was observed as germline variants in the general population (gnomAD and ToMMo) (Table 1).

Since many DD genes overlap oncogenic genes [52], further caution should be appreciated for possible clonal expansion of blood cells, including clonal hematopoiesis with indeterminate potential (CHIP). Among the genes hit by deleterious mDNVs, *ARID2*, *KMT2C*, and *GNAS* are included in the 713 genes associated with cancer in TCGA (https://portal.gdc.cancer.gov/, 2022-05-24) or 87 genes associated with CHIP compiled previously [9]. We formerly confirmed that the mDNV in *KMT2C* was of early developmental origin [9]. We also determined in this study that the LoF mDNV in *ARID2* was of early developmental origin, validating the same variant in the nail DNA (an ectodermal tissue) with a VAF of 4.1% (VAF in saliva = 5.4%). This variant detected in an ectodermal tissue is expected to be shared among various tissues, including the brain. The origin of the variant in *GNAS* remained unknown due to the difficulty of re-contacting. We could not exclude the possibility of CHIP for other mDNVs, but CHIP should not be frequent in 190 BD probands with a median age of 37.0 because CHIP is not frequent before age 40 [60–62]. At least the age-matched 39 controls, including 18 unaffected siblings of BD probands, did not have deleterious mosaic variants in DD genes with similar depth of DES, indicating that deleterious mosaic variants in DD genes were not apparently frequent in healthy controls.

### Mechanistic insight for BD

To obtain mechanistic insight into how mDNVs contribute to the risk for BD, we performed a series of bioinformatics analyses. For this, we used the genes hit by any non-synonymous mDNVs (*n* = 113) rather than those hit by deleterious mDNVs (*n* = 33) to increase statistical power. This expansion was justified because we observed nominal enrichments of DD and ASD genes in the genes hit by non-synonymous mDNVs (*P* = 0.0189 and 0.00519, respectively).

First, we performed gene ontology (GO) enrichment analysis for the genes hit by non-synonymous mDNVs using DNENRICH, which adjusts for gene length and trinucleotide mutational contexts (Table S6). The top-ranked GO was positive regulation of neuron projection development (*P* = 0.000237), which includes three DD/ASD genes (*ARHGAP35, PLXNB1*, and *UNC13A*). This neurodevelopmental GO was consistent with the nominal enrichment of DD/ASD genes in the genes hit by non-synonymous mDNVs. Still, none survived after the adjustment to FDR (FDR < 0.1), probably due to a limited number of the input genes.

We then checked the protein-protein interaction (PPI) networks of proteins coded by the genes with non-synonymous mDNVs in BD. These proteins formed PPI networks more than expected (*P* = 0.0233, background = whole genome). The proteins coded by the DD/ASD genes with non-synonymous mDNVs in BD also formed PPI networks more than expected (*P* = 0.0419, background = 1053 DD/ASD genes), consistent with the nominal enrichment of DD/ASD genes in the genes hit by non-synonymous mDNVs. By visualizing the PPI of genes affected by non-synonymous mDNVs, we found four networks. Of these, the largest network consisting of 22 genes particularly involved many DD/ASD genes, where 11 of them (ARID2, KMT2C, SRCAP, and others) are known to be associated with DD/ASD (Fig. 3C).

### Extensive detection of mosaic variants in non-trio BD cases

In addition to 194 trio-based BD probands, we sought deleterious mosaic variants in the DD/ASD genes from 41 non-trio-based BD cases as an exploratory search (not included in the statistical analysis above). Three cases had deleterious mosaic variants in the DD genes: *CHAMP1*, *NACC1*, and *BCL11B* (Table 1). Another patient had an LoF mosaic variant in an ASD gene: *MED13* (SFARI score 1, pLI 1.00). Yet *MED13* is not in the DD gene list in Kaplanis et al. [52], but was reported to be associated with developmental delay and intellectual disability by autosomal dominant mode, frequently through gDNVs [63]. These data provide additional examples of BD cases with deleterious mosaic variants in the DD/ASD genes, resulting in 9 and 14 individuals with deleterious mosaic variants in DD and ASD genes, respectively, among 231 BD participants.

We observed no deleterious mosaic variants in DD genes but one deleterious mosaic variant in an ASD gene (p.Trp1981* in *LRP1*) in 39 controls of our recruitment. The contrast of deleterious mosaic variant rates of 9/231 (0.039) in BD vs. 0/39 (0.00) in controls for DD genes and 14/231 (0.061) in BD vs. 1/39 (0.026) in controls for ASD genes did not contradict our hypothesis. However, the statistical power was insufficient to assess these enrichments due to a small sample size of controls. For reference, these contrasts indicated *P* values of 0.366 and 0.704 for DD and ASD genes, respectively, by two-sided FET. Further confirmation needs a larger sample size of controls.

### Mitochondrial heteroplasmic variants in BD

**Fig. 4:**
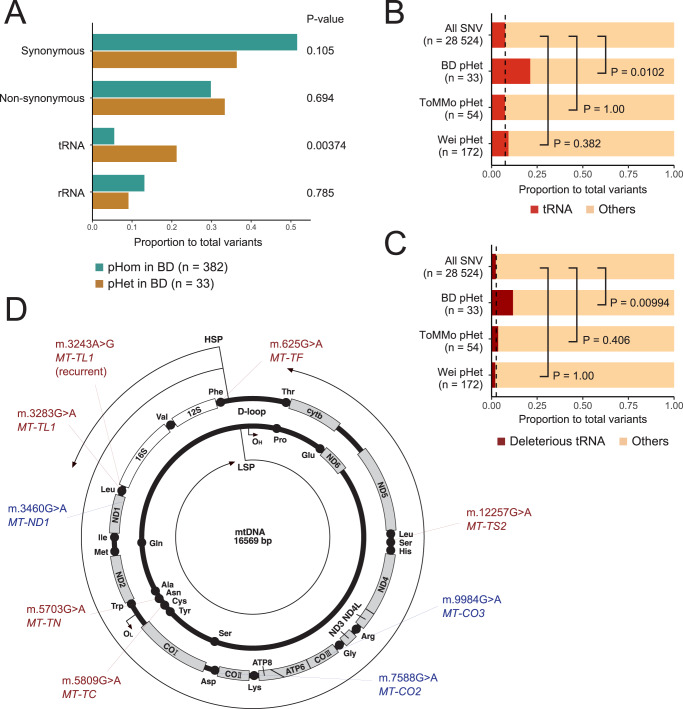
The enrichment of tRNA pHets in BD. **A** The comparison of pHets and pHoms in BD. The x-axis indicates the proportions of each variant class to the total genic variants in the mitochondrial genome. The *P*-values calculated by two-sided FET for the proportion of pHet based on the proportion of pHom are described on the right side. The proportion of tRNA (**B**) and deleterious tRNA (**C**) variants to total variants in four sets: all the possible genic SNVs, pHets in BD, ToMMo, and Wei et al. [55]. The dotted line indicates the proportion of tRNA or deleterious tRNA variants in all the possible genic SNVs. The *P*-values calculated by two-sided FET based on the proportion of all the possible genic SNVs are described on the right side. **D** A schematic view of BD pHets of interest on mitochondrial DNA. Red indicates deleterious tRNA variants, and blue indicates other potentially pathogenic variants (LoF, initiator codon, and ClinVar-pathogenic).

The enrichment of DD/ASD genes in the genes hit by exonic deleterious mDNVs encouraged us to search for similar phenomena in the mitochondrial genome. At a median depth of 711×, we detected 33 high-confident mitochondrial heteroplasmic variants in 190 trio-based BD probands (pHets, Table S7) with VAFs of 1–36% (median 4.1%). The trio-based TAS validation confirmed low heteroplasmic variants (VAF < 10%) by excluding misalignment due to transmitted NUMT (Table S8) (validation rate = 90.7%, all were SNVs). To investigate the characteristics of pHets, we compared the high-confident pHets to homoplasmic variants in the same probands (pHoms, *n* = 382, Table S9), calculating the proportion of four classes of mitochondrial genic variants: synonymous, non-synonymous, tRNA, and rRNA. The proportion of tRNA variants in pHets was higher than in pHoms (*P* = 0.00374, two-sided FET, 3.86-fold enrichment), while the proportions of the other three classes in pHets were not different from those in pHoms (Fig. 4A).

The tRNA variants were more observed as heteroplasmy than homoplasmy in the gnomAD-v3.1 dataset as the general population (Fig. S5A), probably due to selection bias to tRNA variants. The question is whether the enrichment of tRNA variants in BD pHets was derived only from (i) the general feature of heteroplasmy compared to homoplasmy or also derived from (ii) a characteristic of heteroplasmy in BD. The proportion of tRNA variants in the BD pHets was higher than in the gnomAD heteroplasmy (*P* = 0.0226, two-sided FET, 2.42-fold enrichment). However, the gnomAD heteroplasmy included common heteroplasmy, which could have fewer tRNA variants than rare heteroplasmy like those in BD. Thus, we checked the possibility of (ii) by comparing the BD pHets to the newly arising rare pHets in two population datasets: ToMMo GenII [57] and Wei et al. [55]. Since the sequencing methods and analytical pipelines were different, we compared the proportion of tRNA pHets to the total pHets in each set with theoretical proportion (i.e., all the possible mitochondrial genic SNVs). While the proportion of tRNA pHets in the two population datasets is not higher than in all the possible genic SNVs (*P* = 1.00 and 0.382 for ToMMo GenII and Wei et al. data, respectively, two-sided FET), the proportion of tRNA pHets in BD was higher than in all the possible genic SNVs (Fig. 4B, *P* = 0.0102, two-sided FET, 2.82-fold enrichment). The enrichment of tRNA pHets in BD remained significant when limiting the tRNA pHets to the deleterious ones (Fig. 4C, *P* = 0.00994, two-sided FET, 4.72-fold enrichment). These enrichments were not likely to be derived from the bias of SNV patterns in tRNA regions because the SNV patterns in tRNA regions were similar to those in whole mitochondrial genic regions (cosine similarity = 0.894, Fig. S5B). Thus, the enrichment of tRNA variants in BD pHets could be derived not only from the general feature of heteroplasmic variants but also from a characteristic of BD. Besides high-confident variants, we detected two more low-confident heteroplasmic tRNA variants in low-mappability regions in 190 trio-based BD probands, including the ClinVar-Pathogenic m.5703 G > A variant (not included in the statistical analysis above, Table S7).

While we could not statistically assess the enrichment of LoF variants due to the limited number, one participant had an LoF pHet (p.Gly260* in *MT-CO3*). Mitochondrial LoF variants are almost none as homoplasmy in large-scale databases [43, 44]), indicating phenotypic disadvantages resulting from mitochondrial LoF variants. Another interesting variant was an initiator pHet (c.3 G > A) in the AUG start codon of *MT-CO2*. The c3.G > A in the AUG start codon is not observed as homoplasmy in gnomAD and HelixMTdb [43, 44]. In theory, the LoF and AUG initiator-codon pHets could have a pathological effect on BD.

### Extensive detection of heteroplasmic variants in BD

As an extensive exploratory analysis, we sought heteroplasmic tRNA variants, including those in low-mappability regions, from 231 BD and 39 controls, irrespective of transmission status. We found 15 individuals with heteroplasmic tRNA variants in 231 BD participants, including nine tRNA variants as trio-based pHets (Table S7). In contrast, we found no heteroplasmic tRNA variants in 39 controls by the same pipeline (15/231 [0.065] vs. 0/39 [0.00]). Despite the insufficient statistical power due to limited controls (*P* = 0.139 by two-sided FET) and the low-mappability of several variants, this observation did not contradict the possible enrichment of heteroplasmic tRNA variants in BD. Among them, seven deleterious tRNA variants were notable for their rarity in the general population (Table 2). Combining the above results, the heteroplasmic variants of interest for potential pathogenicity (deleterious tRNA, LoF, initiator codon, or ClinVar-registered) are illustrated and listed in Fig. 4D and Table 2.

**Table 2:** The heteroplasmic variants with potential pathogenicity in BD.

Family ID	Diagnosis	Variant	Type	Gene	Proband VAF (%)	MitoTIP^a^	PON-mt-tRNA^a^	ClinVar		Homoplasmy			Heteroplasmy		
								Pathogenicity	Disease	gnomAD	ToMMo	Helix	gnomAD	ToMMo	Helix
**Trio-based**															
523	BDI	m.625 G > A	tRNA	*MT-TF*	4.24	LP	LP	.	.	0	0	0	5.32 × 10^-5^	0	0
404	BDI	m.3243 A > G	tRNA	*MT-TL1*	12.06	PP	LP	Pathogenic	MELAS	0	0	1.02 × 10^-5^	0.00101	0.00130	0.000250
524	BDII	m.3243 A > G	tRNA	*MT-TL1*	5.24	PP	LP	Pathogenic	MELAS	0	0	1.02 × 10^-5^	0.00101	0.00130	0.000250
467	BDII	m.5703 G > A	tRNA	*MT-TN*	12.63	LP	LP	Pathogenic	Ophthalmo-plegia	0	0	0	0.000355	0.000260	2.55 × 10^-5^
423	BDI	m.7588 G > A	initiator p.Met1?	*MT-CO2*	1.6	.	.	.	.	0	0	0	0.000213	0	5.10 × 10^-6^
132	BDI	m.9984 G > A	nonsense p.Gly260*	*MT-CO3*	8.7	.	.	US	Leigh	0	0	0	0.000603	0	3.06 × 10^-5^
435	BDII	m.12257 G > A	tRNA	*MT-TS2*	1.41	LP	LP	.	.	0	0	0	7.09 × 10^-5^	0	0
**Non-trio-based**															
567	SCZAD Bipolar type	m.3283 G > A	tRNA	*MT-TL1*	1.1	PP	LP	.	.	0	0	0	3.54 × 10^-5^	0	0
556	BDI	m.3460 G > A	missense p.Ala52Thr	*MT-ND1*	1.2	.	.	Pathogenic	MCID	0	0	0	0.000160	0.000260	7.14 × 10^-5^
559	BDI	m.5809 G > A	tRNA	*MT-TC*	3.1	PP	P	US	MELAS	5.32 × 10^-5^	0.0001	8.16 × 10^-5^	0.000177	0	4.08 × 10^-5^

*Leigh* Leigh syndrome, *MCID* Mitochondrial Complex I Deficiency, *MELAS* Mitochondrial myopathy, Encephalopathy, Lactic Acidosis, and Stroke-like episodes, *US* Uncertain Significance.

^a^*LP* Likely Pathogenic, *PP* Possibly Pathogenic, *P* Pathogenic, *LN* Likely Neutral.

### Recurrent m.3243 A > G variants in BD

In this study, the m.3243 A > G variant (deleterious tRNA variant) was the only mosaic variant recurrently found in two unrelated BD probands. The m.3243 A > G variant is causative for severe mitochondrial disease, MELAS (mitochondrial myopathy, encephalopathy, lactic acidosis, and stroke-like episodes) [64]. The VAFs of m.3243 A > G in BD probands (5–12%) were lower than the average VAFs in MELAS pedigrees (around 20% in the leucocytes [65]), suggesting a possible correlation between VAFs and disease severities. The m.3243 A > G variant of the ID524 patient was assumed to be de novo, while the m.3243 A > G variant of ID404 was inferred as maternally inherited with low-level heteroplasmy because the mother of ID404, diagnosed with recurrent depression, had the m.3243 A > G variant with a VAF of 2.0%. The two pedigrees seemed genetically unrelated, because the PHI score between the two BD probands was −0.0147 by vcftools-v0.1.17 relatedness2 [66, 67] calculation of exonic common SNPs. The pedigree of ID404 had two other psychiatric patients with schizophrenia in the mother’s pedigree compatible with maternal inheritance. While the DNA samples from the two schizophrenia patients and systematic pedigree information were unavailable, this information motivated us to investigate pHets in schizophrenia. Selecting 143 schizophrenia trios with the probands’ average mitochondrial depth ≥50 as eligible from Fromer et al. data [50], we detected 4 pHets in the 143 trios, including one m.3243 A > G variant with a VAF of 13.3% in one schizophrenia proband (frequency = 6.99 × 10^−3^).

Then, we queried if the m.3243 A > G variant is frequent in large-scale population datasets (Table S10). First, we investigated Japanese population samples, ToMMo-8.3KJPN (median mitochondrial depth of 786×) [33, 56]. The m.3243 A > G variants with VAFs ≥ 1% were detected in 10 of 7720 individuals (frequency = 1.30 × 10^−3^). The proportion of individuals with m.3243 A > G variants in BD (2/231 = 8.66 × 10^−3^) was higher than in ToMMo-8.3KJPN with similar depths of around 750× (*P* = 0.0464, two-sided FET). Next, we checked the gnomAD v3.1 database and found 57 m.3243 A > G variants (VAFs ≥ 1%) in 56 383 individuals, including ambiguous calls of VAFs < 10% [43]. Since the median mitochondrial depth of 2700× in gnomAD-v3.1 indicates sufficient sensitivity to VAF of 1%, we can safely interpret the upper estimate of the frequency of m.3243 A > G variants with VAFs ≥ 1% as 1.01 × 10^−3^ in gnomAD-v3.1. Even using this upper estimate, the proportion of individuals with m.3243 A > G variants in BD was higher than in gnomAD-v3.1 (*P* = 0.0247, two-sided FET). We also checked m.3243 A > G frequency in HelixMTdb [44], an investigation using saliva DNA like our BD study, and found the frequency of m.3243 A > G variant as 2.60 × 10^−4^ at an average mitochondrial depth of 182×. We queried the downsampled (to 182×) data of BD to match the source tissue (saliva) and sequencing depth between BD and HelixMTdb, confirming that the two pHets of m.3243 A > G in BD were still callable at 182× depth. The proportion of individuals with m.3243 A > G variants in BD was also higher than in HelixMTdb (*P* = 0.00186, two-sided FET) in the comparison matching the source tissue and sequencing depth. Thus, the frequency of m.3243 A > G variants in BD should be higher than in the three population datasets.

## Discussion

Here, we report the characteristics of exonic and mitochondrial mosaic variants in BD comprehensively detected by DES using peripheral tissues. The genes hit by deleterious mosaic variants in BD were enriched in DD/ASD genes (Fig. 2). These exonic deleterious mosaic variants were not observed in the general population (Table 1). The PPI network analysis suggested that BD has shared pathological mechanisms with DD/ASD through mosaic variants (Fig. 3). The mitochondrial heteroplasmic variants in BD probands were enriched in tRNA regions (Fig. 4), including recurrent m.3243 A > G variants, and the deleterious tRNA variants in BD were rare in the general population (Table 2).

While using peripheral tissues, the enrichment of DD genes in the genes hit by deleterious mDNVs in BD (Fig. 2) supports our primary hypothesis that deleterious mosaic variants in DD genes are associated with BD. Post hoc power calculation indicates that our sample size is enough to address this issue, and deleterious mosaic variants in DD genes were not observed in DES from 39 control samples. Our data extend Sanders et al.’s perspective on the relevance of rare genetic diseases to neuropsychiatric symptoms [68]: the deleterious mosaic variants in DD genes would result in less severe phenotypes (e.g., BD) as a biological spectrum from DD (Fig. S6). The mosaic LoF variant in *ARID2* in BD, which causes Coffin-Siris syndrome if it exists as a germline variant, is a notable example of this paradigm. As a more general pathological insight, mosaic variants in DD/ASD genes could be one of the molecular explanations behind the shared molecular pathologies among various psychiatric/neurodevelopmental disorders [69]. Indeed, the PPI of the proteins coded by the DD/ASD genes with non-synonymous mDNVs in BD, especially the tight interactions of ARID2, KMT2C, and SRCAP as chromatin remodeling proteins, should be an exemplar shared molecular mechanism between BD and DD/ASD.

We also found the potential relevance of mitochondrial heteroplasmic (mosaic) variants to BD. The enrichment of tRNA heteroplasmic variants, especially those with predicted deleterious effects, suggests that tRNA variants could explain a part of BD. The enrichment of heteroplasmic tRNA variants in BD was calculated by the proportions of tRNA to total variants in our study. Assuming the mutation rate of mtDNA as a molecular property in BD is no less than in population controls, the tRNA variants would be more observed in BD than in population controls. Wang et al. reported that tRNA heteroplasmic variants are more observed in ASD than in unaffected ASD siblings, while the overall heteroplasmic variant rates were not different [70]. This contrast could also be applicable to BD. Indeed, our preliminary direct comparison suggests enrichment of heteroplasmic tRNA variants in BD qualified with a limited sample size of controls (BD vs. control = 15/231 [0.065] vs. 0/39 [0.00]). Mitochondrial dysfunction has been hypothesized to explain BD [71], particularly BD with maternal inheritance [72]. Since mitochondria have critical roles in brain function as energy sources of ATP and calcium ion regulators, mitochondrial dysfunction should result in alterations in brain function, including psychiatric symptoms [73]. Indeed, patients with mitochondrial diseases tend to have psychiatric symptoms, including BD [74–76]. Especially, mitochondrial disease patients with m.3243 A > G variants are more susceptible to BD type I than the general population (odds ratio = 12.8) [76]. Brain-specific *Polg* mutant mouse, in which various mitochondrial DNA variants are accumulated in their brains due to mitochondria-specific DNA polymerase gamma impairment, shows recurrent mood disorder-like phenotypes, including antidepressant-induced manic-like behavior [77]. Here, we found the enrichment of a specific class of mitochondrial DNA variants as a potential background for BD. The question is whether the heteroplasmy with lower fractions than mitochondrial diseases has a pathogenic effect. Some thresholds of VAFs should exist at the cellular level, but the thresholds for psychiatric disorders are not sure, which could be addressed in future molecular studies. Technically, we could detect low heteroplasmic variants with trio-based validation by removing false positives due to misalignment by NUMT, which were otherwise difficult to confirm without trios. This method could be used in future mitochondrial analysis to detect low heteroplasmic variants precisely.

Of note, we found recurrent m.3243 A > G variants from two unrelated BD probands and two patients with other psychiatric disorders, schizophrenia, and recurrent depression. The higher frequency of the m.3243 A > G variant in BD than in the general population suggests a possible association of the m.3243 A > G variant with BD. Indeed, mitochondrial disease patients with m.3243 A > G variants are more likely to have BD than the general population [76]. Our observation of the m.3243 A > G variants in BD and schizophrenia is consistent with Munakata et al. [78], in which the m.3243 A > G variants were found in two and one brains of 15 BD and 13 schizophrenia patients, respectively (VAFs of 1~2%), but none in the brains of 14 healthy controls. Low heteroplasmic m.3243 A > G variants might be observed frequently in the brains as a trans-diagnostic background of major psychiatric disorders. The pathogenicity of the m.3243 A > G variant is well established for mitochondrial disease, MELAS [64], which is much more severe than BD and schizophrenia. Lower VAFs of m.3243 A > G in BD and schizophrenia than MELAS indicates a possible phenotypic spectrum by VAFs: low heteroplasmic variants, which would result in mitochondrial diseases with high VAFs, should result in milder phenotypes, including psychiatric symptoms (Fig. S6). Indeed, high VAFs of m.3243 A > G variants are related to severe neurological symptoms [79]. One study using neurons differentiated from induced pluripotent stem cells from patients with the m.3243 A > G variant reported that the neuronal dysfunctions from m.3243 A > G follow the VAFs (e.g., more than 30% of mutant alleles lead to neuronal burst irregularity in vitro) [80]. We can speculate that heteroplasmy levels exceed the threshold only in a subgroup of neurons in our BD probands, resulting in psychiatric symptoms related to BD. If this holds, a new therapeutic strategy of taurine for MELAS [81] is a potential therapeutic repurposed for psychiatric patients with m.3243 A > G. Other treatment strategies targeting mitochondrial dysfunctions, especially those derived from m.3243 A > G [82, 83], are also potential therapeutics for psychiatric patients. In parallel with drug development, genomic stratification could be a promising path to precision medicine in psychiatry.

Despite the findings above, we are aware of the following limitations. First, the enrichment of DD/ASD genes in the genes hit by deleterious mDNVs in BD was based on a theoretical estimate. While we confirmed that this enrichment was not observed in gDNVs in BD and mDNVs/gDNVs in unaffected siblings in ASD studies, future direct case-control comparisons will provide further conclusive results. Second, this study detected the mDNVs in BD in peripheral tissues. We validated two critical mDNVs in *ARID2* and *KMT2C* as of early developmental origin, but a possible clonal expansion of blood cells cannot be completely excluded for other mDNVs. Direct investigation of the human brain is desirable for psychiatric research. Besides, our recruitment of BD participants would cause ascertainment bias among BD patients. They were voluntarily interested in scientific research as a family and could have different intellectual capacities from general BD patients. These limitations are also applicable to our analysis of mitochondrial DNA variants. The heteroplasmy in BD also needs case-control comparison and direct investigation of the brain samples. Brain samples are more required for mitochondrial analysis because mitochondrial heteroplasmy is highly variable across tissues. We used various datasets with different source tissues and depths to assess the enrichment of m.3243 A > G heteroplasmy in BD, but the exact frequency of m.3243 A > G heteroplasmy should be investigated in a research design matching the source tissues and sequencing depths.

The use of peripheral tissues is a major limitation of our study. We did not target brain-specific mosaic variants, which could contribute to BD. The mosaic variants in the brain were enriched in the genes involved in neural function with a bias to exonic regions [84, 85]. These variants should be targeted in future investigations. However, we emphasize that peripheral tissues have potential clinical significance. While it is true that postmortem brain samples are the best material for studying mosaic variants directly involved in the pathogenesis of BD, the brains of living patients are usually inaccessible. In contrast, peripheral tissues could be used for clinical genomic diagnosis and risk prediction. Besides, the high accessibility of peripheral tissues enables large-scale case-control comparisons and studies of rare diseases. The possibility of mosaic variants due to clonal hematopoietic expansion can be excluded by assaying other tissues (e.g., nails) as performed in our study. We extensively discuss this issue in Supplementary Note.

Another limitation is the functional prediction of exonic variants. We used canonical transcripts for annotation with the “SnpEff -canon” argument (Supplementary Methods) as a fair analysis as much as possible. However, the complex alternative splicing patterns in the brain could be relevant to the mechanisms of psychiatric disorders [86, 87]. The correct functional annotations for brain-expressed genes need further sophistication that should be addressed in future studies.

In conclusion, we find the enrichment of DD/ASD genes in the genes hit by deleterious mosaic variants in BD with certain statistical evidence, supporting the primary hypothesis of this study. We also find the enrichment of mitochondrial tRNA heteroplasmic variants in BD, including recurrent m.3243 A > G. The m.3243 A > G variants are more observed in BD than in the general population and detected with lower allele fractions than mitochondrial diseases, indicating a possible phenotypic spectrum following variant allele fractions. Since our study has the limitation of peripheral tissue and insufficient control samples, future well-powered case-control investigations into brain samples are required. Despite these limitations, our data shed new light on the genetic architecture of BD and pave the way for precision medicine in clinical psychiatry by genomic stratification.

### Supplementary information

Supplementary Information

Dataset 1

Dataset 2

Dataset 3

Dataset 4

Dataset 5

## Data Availability

The conventional WES data with consent for database registration are available through the NBDC Human Database, Japan (JGAS000273/JGAD000379). The deep exome sequence data can be accessed via formal collaboration.
