# Supplementary material for: Deep exome sequencing identifies enrichment of deleterious mosaic variants in neurodevelopmental disorder genes and mitochondrial tRNA regions in bipolar disorder
Source: Mol Psychiatry. 2023 May 30;28(10):4294–306. doi: 10.1038/s41380-023-02096-x (PMC10827672; doi:10.1038/s41380-023-02096-x)
Supplement: Supplementary file 1 — Supplementary Information [file 41380_2023_2096_MOESM1_ESM.pdf]

Supplementary Information

Supplementary Methods **2**

Study Participants 2

Deep exome sequencing 2

Read alignment and variant calling 3

Variant annotation 5

Validation of mosaic variants 5

Referential variant datasets from ASD studies 6

The enrichment analysis of mDNVs in BD 6

Gene ontology analysis and protein-protein interaction enrichment 7

Heteroplasmic and homoplasmic variant comparison in BD probands 8

Heteroplasmic variants in other datasets 8

Statistical Analysis 9

Data availability 9

Supplementary Note **11**

Methodological note on mosaic variant detection using peripheral tissues 11

Supplementary Figures **14**

Figure S1. The mutation patterns and VAFs of mDNVs in BD 14

Figure S2. The enrichment of DD/ASD genes in the genes hit by mDNVs/gDNVs in ASD 15

Figure S3. DNENRICH analysis of LoF variants 16

Figure S4. The amino acid conservation around the damaging missense mDNVs 17

Figure S5. The mitochondrial variant analysis related to Figure 4 18

Figure S6. A model of phenotypic spectrum by mosaic variants 19

Supplementary Methods

Study Participants

We recruited 194 BD probands with their parents (trios), 41 BD single cases, and 39 controls without psychiatric disorders, including 18 unaffected siblings of BD probands. The participants with BD and their parents (trios) were recruited through Bipolar Disorder Research Network Japan (http://bipolar.umin.jp/) and the participating institutions. The BD participants were clinically diagnosed with BD or schizoaffective disorder (SCZAD) by trained psychiatrists. Their diagnoses were formally verified based on DSM (Diagnostic and Statistical Manual of Mental Disorders) -IV, -IV-TR, or -5, using a structured or semi-structured interview [10] by a psychiatrist. All the parents were screened for mental disorders by a structured interview [10], except for some cases with communication disabilities for whom interviews were done using printed matter. After a detailed explanation and obtainment of written informed consent, the saliva or blood of the participants was collected.

Among the 235 BD participants, 131 overlapped with our previous study [9], and 104 were newly recruited. The participants with parents with BD, SCZAD, or schizophrenia (N of trios = 9) and the participants with few remained DNA (N of trios = 31) in our previous study were excluded from this study. The average age at recruitment of BD and control participants is 36.7 ± 9.33 (standard deviation) and 36.9 ± 6.5, respectively. The average ages of BD and control were not statistically different ($P = 0.910$, two-sided Welch's t-test). The average age at disease onset of BD participants is 24.6 ± 8.32. The saliva samples were collected by Oragene DISCOVERY kit (DNA Genotek, Ottawa, Ontario, Canada). This study was designed according to the Helsinki declaration and approved by the Research Ethics Committee, Faculty of Medicine, Juntendo University, RIKEN Wako Research Ethics First Committee, the Ethics Committee on Genetics of Niigata University, and the Research Ethical Committee of Tohoku Medical Megabank Organization, Tohoku University.

Deep exome sequencing

All the DNA samples of 235 BD participants and 39 controls newly underwent DES, including the 131 overlapped BD samples from our previous study [9]. The DNA from the parents of new trios underwent conventional WES. The exonic and mitochondrial DNA was enriched by Agilent SureSelect Human All Exon v5/6 kits and custom mitochondrial DNA probes (Agilent Technologies, Santa Clara, CA, USA) according to the manufacturer's

protocol. The prepared library underwent deep sequencing by NovaSeq6000 or NextSeq2000 (Illumina, San Diego, CA, USA) with 151 bp pair-end cycles to aim for a theoretical depth of 500×. To obtain higher depth, the DES reads of the overlapped 131 trios were combined with the previous conventional WES data [9]. One BD proband was excluded from the subsequent analysis due to the low depth of coverage (< 100×). The phenotypes and sequencing data for each participant are summarized in **Table S1**.

Read alignment and variant calling

We used the BWA-GATK-based pipeline as in our previous study [9] with the two different human reference genomes (hg38 and hs37d5) for extensive variant discovery. The detailed pipeline is described in **Table S2**. Briefly, we aligned the raw sequence reads to the reference genomes by BWA-0.7.17 [26] and performed the subsequent quality control by GATK-4.0.12.0 according to the GATK Best Practice workflow [27,28]. The candidate mosaic variants were called by Mutect2 [29] in GATK-4.1.0.0. We called the candidate exonic and mitochondrial mosaic variants in the trio-based procedure by Mutect2 to filter out transmitted germline variants and frequently observed sequencing artifacts. The parental BAM files were used as normal samples (-normal) for the probands' BAM files (-tumor) in the Mutect2 analysis. We used the father's sequencing data for mitochondrial variants to exclude false positives derived from partial misalignment to transmitted nuclear mitochondrial DNA (NUMT) [47]. The sequencing data from 41 non-trio-based BD cases without the parental sequence data and 39 controls underwent Mutect2 calling without a "-normal" parameter as an exploratory analysis. The mitochondrial heteroplasmic variants in the exploratory analysis were called by Mutect2 with the --mitochondria-mode argument. We prepared our Panel of Normals (PoN) for the Mutect2 pipeline from a cohort of control samples without a diagnosis of BD, SCZAD, or schizophrenia (N = 723, including the parents in our previous study [9] and unrelated in-house controls, **Supplementary Data**). We also applied the standard PoN provided by Broad Institute to our pipeline. The PoNs filtered out the genomic positions that harbor frequently observed artifacts (position-based). We also filtered out the variants reported as non-PASS in gnomAD r2.1.1 [30] as probable sequencing artifacts (allele-based). The same filtering was applied to the candidate calls from BD cases and controls.

We selected exonic mDNVs with the following parameters: (i) variant allele fraction (VAF) of the proband < 0.40 and > 0.01, (ii) allele count of the proband ≥ 7, (iii) variant calls both on forward and reverse reads ≥ 2, (iv) allele count of each parent ≤ 1, (v) depth of each parent ≥ 10, (vi) TLOD (fidelity score by GATK-4.1.0.0 Mutect2) ≥ 15, (vii) average

mapping quality (MQ) = 60 (maximum value in our pipeline), and (viii) the probability of germline variants as bcftools PBINOM(AD) > 20 and GERMQ in Mutect2 > 200. The mitochondrial heteroplasmic variants in the probands (pHets) were selected with the following parameters: (i) VAF of the proband < 0.90 and > 0.01, (ii) allele count of the proband ≥ 5, (iii) variant calls both on forward and reverse reads ≥ 2, (iv) allele count of each parent ≤ 1, (v) depth of mother ≥ 5, (vi) TLOD ≥ 15, and (vii) average MQ > 40. We set more relaxed thresholds for mitochondrial variants than exonic variants according to the result of pilot validation experiments as described below. We classified the pHet into two categories: high confidence (average MQ = 60) for statistical analysis and low confidence (average MQ < 60) for exploratory analysis. We comprehensively investigated the mDNVs and pHets in the trio-based BD probands but did not in the non-trio-based BD cases and controls. Comprehensive discovery of mDNVs and pHets without the parental data is challenging due to the abundance of the candidate mosaic variants in the non-trio-based samples. We limited the use of the non-trio-based BD cases and controls to exploratory analysis.

For trio-based BD probands, the outliers in per-individual mDNV counts that deviated from the expected Poisson distributions were excluded from the subsequent analysis (N = 3). Outliers were defined by using the *dpois* function of R as follows: case α_i with mDNV count x_i was considered as an outlier if dpois(x_i, lamda=*u*)**n* was less than 0.05, where *u* is an average mDNV count and *n* is a total mDNV count including case α_i. This procedure was performed iteratively from the case with the maximum mDNV count. As a result, three samples were outliers, and the remaining 190 trio-based probands were the target of statistical analysis. This procedure did not apply to the BD cases without parental sequence data.

The germline de novo variants (gDNVs) in the same trio-based BD probands (N = 190) were partly compiled from our previous study [9] and additionally called by the same procedure as in our previous study [9] for the newly recruited trios (**Table S2**). Briefly, we called all the germline variants by HaplotypeCaller, GenomicDB, and GenotypeGVCF of GATK-4.0.12.0/4.1.0.0. The gDNVs were selected by triodenovo-0.06 [31] and DNMFilter-0.1.1 [32] with a threshold score of 0.6. The mitochondrial homoplasmic variants in the BD probands (pHoms) were called by HaplotypeCaller, GenomicDB, and GenotypeGVCF of GATK-4.0.12.0, setting the ploidy as 1. We selected the high-confident variants using the following parameters: MQ = 60, QD ≥ 5, and SOR < 3. Since the analytical engine of HaplotypeCaller is shared with Mutect2 in GATK, the calling bias should be consistent between the mDNVs and gDNVs or between pHets and pHoms in BD. The variants detected by the hs37d5 pipeline underwent liftover to the

hg38 coordinate for variant annotations.

Variant annotation

The exonic variants were annotated with public data and *in silico* prediction software as follows: allele frequency in gnomAD-r2.1.1 in all (N = 125 748) or non-neuropsychiatric samples (N = 104 068) [30], allele frequency in ToMMo-8.3KJPN [33] (N = 8380), effect on protein function predicted by SnpEff-4.3 [34] (GRCh38.86 -canon), pathogenicity inference by MPC [35], and predicted effect on protein function of missense variants by dbNSFP-4.0a [36]. Seven algorithms for functional prediction of missense variants in dbNSFP (SIFT [37], PolyPhen-2 HumVar and HumDiv models [38], LRT [39], MutationTaster [40], Mutation Assessor [41] and PROVEAN [42]) were adopted from our previous study of BD [9]. The most severe effect was annotated if the prediction outputs two or more effects (depending on the number of transcripts) for one variant. Loss-of-function (LoF) variants include nonsense, frameshift, and canonical splice variants. Damaging missense variants in this study mean missense variants with MPC ≥ 2 or predicted as damaging by all the seven algorithms in dbNSFP annotation. We defined deleterious variants as LoF or damaging missense variants.

The mitochondrial variants were annotated with public data and *in silico* prediction software as follows: allele frequency in gnomAD-v3.1 [43] (N = 56 434), allele frequency in ToMMo-8.3KJPN [33] (N = 8380), allele frequency in HelixMTdb [44] (N = 195 983), effect on protein function predicted by SnpEff-4.3 [34] (GRCh38.86 -canon with mitochondrial configuration). The effects of tRNA variants followed the prediction by MitoTIP [45] and PON-mt-tRNA [46]. The deleterious mitochondrial tRNA variant was defined as those with a MitoTIP score > 12.66 and PON-mt-tRNA score > 0.5, adopting the original threshold.

All these items were annotated in the hg38 coordinate. The ClinVar annotation was derived from file: clinvar_20210524_GRCh38.vcf.gz. The variants from other publications were reannotated with the same procedure.

Validation of mosaic variants

The candidates of mosaic variants underwent validation experiments of target amplicon sequencing (TAS) by MiSeq or iSeq100 (Illumina) with 151 cycles pair-end mode. The samples were indexed using the conventional indexing and the initial six bases in the first read to exclude the sample cross-contamination. For pHets, the candidate variants underwent TAS for the proband, mother, and father to exclude the possibility of misalignment due to NUMT transmission. This approach enabled us to detect low

heteroplasmic variants precisely by removing false positives due to misalignment by NUMT, which otherwise are difficult to confirm. The low heteroplasmic variants (VAF < 10%) in gnomAD were labeled as ambiguous calls without parental data [43].

We designed PCR primers for candidate sites that yielded single-banded PCR amplicons of expected sizes. Samples with poor DNA quality due to long storage were omitted in the validation experiment. Sequencing libraries for TAS were prepared by two rounds of PCRs and analyzed as previously described [9,48,49]. Briefly, the sequence reads were aligned to hg38 by BWA-0.7.17 (median 26 719×). We counted the base calls with base quality ≥ 30 and MQ = 60 for the mosaic variants, then calculated the VAF for total base calls on the candidate sites. The candidates with the allele fraction ≥ 0.3% (assumed sequencing error rate) by TAS analysis were considered validated. The non-synonymous variants were prioritized for mDNV validation. For pHets, we also counted the base calls with MQ ≥ 0, 20, or 40 to accommodate the variants in low-mappability regions due to NUMT. The mitochondrial variant with MQ < 60 in TAS was confirmed as non-NUMT if the VAFs in the parents were below 0.3%, including MQ = 0 reads. We limit the pHets for the subsequent analysis to those validated by TAS except for one trio with poor DNA quality (ID406). The variants failing in TAS validation were excluded from the subsequent analysis.

Referential variant datasets from ASD studies

We compiled the referential gDNV/mDNV sets in ASD probands and unaffected ASD siblings as positive and negative controls, respectively, from previous studies [18,51]. We limited the comparison to single nucleotide variants (SNVs) because insertions/deletions (INDELs) detection is sensitive to pipeline differences. The gDNVs in ASD probands (n of gDNVs = 7623, N of trios = 6430) and unaffected ASD sibling (n of gDNVs = 2197, N of trios = 2179) samples were compiled from Satterstrom et al. [51]. The mDNVs in ASD probands (n of mDNVs = 320, N of trios = 2264) and unaffected ASD sibling (n of mDNVs = 202, N of trios = 1698) samples were compiled from Krupp et al. [18], selecting high confident mDNVs with PHET_MAIN < 0.01. The variants underwent the matching annotation procedures for BD.

The enrichment analysis of mDNVs in BD

We assessed the enrichment of DD and ASD genes in the genes hit by mDNVs and gDNVs in BD, ASD probands, and unaffected ASD siblings by DNENRICH [50] with one million random permutations. DNENRICH is a statistical package to calculate the probability of the observed gene count by permutation, adjusting per-gene mutation rates by gene

length and trinucleotide contexts (both influence per-gene mutation rates). The background genes were 19,637 canonical protein-coding genes in the SnpEff GRCh38.86 database. The DD genes (285 genes) were defined as DD-associated genes through gDNVs in Kaplanis et al. [52], the most extensive gDNV study for DD to date. The ASD genes (924 genes) were defined as genes with SFARI scores 1–3 (file: SFARI-Gene_genes_01-13-2021release_06-02-2021export.csv, https://gene.sfari.org/). Before using DNENRCH, originally designed for gDNV analysis, we examined whether the mutation spectrum of the SNVs in mDNVs did not differ from the mutation spectrum of the SNVs in gDNVs. For comparison, all the six variant sets (mDNVs/gDNVs in BD/ASD/ASD sibling) underwent the same position-based filtering (i.e. our original PoN + Broad PoN [position-based] and gnomAD nonPASS panel [allele-based]). The mDNVs and gDNVs in BD were derived from the same cohort of BD probands, and the analytical engine is shared between Mutect2 and HaplotypeCaller in GATK. Thus, we assumed that the variant calling bias in the mDNVs and gDNVs in BD was not different. Since the variant calling pipelines of the mDNVs/gDNVs in ASD/ASD siblings differed from our pipeline, we used these datasets as the positive/negative control sets (not directly comparable) to check the validity of the DNENRICH analysis.

In addition to the above DNENRICH analysis, we analyzed DES data from 39 controls of our recruitment, including 18 unaffected siblings of BD probands, to rule out the apparent technical artifacts and observed clonal hematopoietic expansion. The average depths of DES from BD and control were not statistically different ($P = 0.605$ for hg38; $P = 0.680$ for hs37d5, two-sided Welch's t-test, **Table S3**). Due to the relatively small sample size, the primal purpose of this analysis was not to compare the BD cases and controls with sufficient statistical power but to check frequently observed technical artifacts and variants from clonal hematopoietic expansion in control samples.

Gene ontology analysis and protein-protein interaction enrichment

We performed gene ontology (GO) enrichment analysis by DNENRICH [50] with one million random permutations with the same background genes described above, using the PantherGO-slim (PantherGOslim.obo, http://www.pantherdb.org/) as the GO term sets. DNENRICH adjusts per-gene mutation rates considering gene length and trinucleotide contexts, both of which influence per-gene mutation rates. The geneset in each GO term for DNENRICH is the same as in our previous study [9] (**Supplementary Data**). We excluded the GO terms including a small number (< 30) of genes, which have limited statistical power, and the terms including a vast number (> 1500) of genes, which are not informative when exploring specific pathways. The false discovery rate (FDR) was

calculated separately for three categories of GO (biological process, molecular function, and cellular component), and < 0.1 was set as significant as previously [9]. We used STRING [53] for protein-protein interaction (PPI) analysis (https://string-db.org/, analysis on 2022-05-24). Network visualization of the result of STRING analysis was done by Cytoscape v3.7.2 [54]. The clusters with three or fewer proteins were omitted for simplicity.

Heteroplasmic and homoplasmic variant comparison in BD probands

We classified the mitochondrial genic variants into four classes: synonymous (protein-coding), non-synonymous (protein-coding), tRNA, and rRNA. The intergenic regions (mainly D-loop) were not the target of our study due to much higher mutation rates than genic regions [55]. We compared the proportions of the variants in each class to the total mitochondrial genic variants in the pHets and pHoms from the same BD trios (N = 190). We regarded the pHets and pHoms were comparable because Mutect2 (for pHets) and HaplotypeCaller (for pHoms) share the same analytical engine in GATK, having matching calling biases. We limited the comparison to SNVs and filtered out the mitochondrial variants in low-mappability regions defined as follows (5188 bp [31.3%], **Supplementary Data**). First, we extracted a 301-bp-long sequence from the reference hg38 mitochondrial sequence with the name of the center position (i.e., each center position has 150 bp extensions for both directions). Second, we aligned the extracted 301-bp-long sequence to hg38. The MQ of the sequence was calculated, and lower than 60 means that the original center position is difficult to precisely map by current short-read technology. 150 bp was set as the read length of our study. The pHets and pHoms underwent the same position-based filtering (i.e., our original PoN, Broad PoN, and low-mappability filter: 6179 positions [37.3%] are excluded in these filters).

Some heteroplasmic variants are potentially transmitted from the mother; others are de novo. The transmission status of mitochondrial heteroplasmic variants is inherently difficult to resolve due to the high variability of VAFs in one's life course. Heteroplasmic variants can increase or decrease with age, occasionally to zero. Thus, we operationally defined the pHet as probands' heteroplasmic variant as described above.

Heteroplasmic variants in other datasets

We compared the pHets in BD to all the possible genic variants in the mitochondrial genome by calculating the proportions of the target variants to the total genic variants. The analysis was limited to SNVs not in low-mappability regions. We also checked the characteristics of pHets in two population datasets as references for BD: ToMMo

[33,56,57] and Wei et al. study [55]). ToMMo dataset is whole-genome sequencing (WGS) data collection of the general Japanese population aligned to hs35d5, which shares the mitochondrial sequence with hg38. We called the pHets in the ToMMo trio generation II (ToMMo GenII, N = 518 trios, median depth of the offspring = 827×) [57] with the matching Mutect2-based procedure for BD trios. The average age of ToMMo GenII (32.6 ± 4.9) is near the BD cohort's average age. The frequencies of the target heteroplasmic variants (e.g., m.3243A>G) in the ToMMo-8.3KJPN panel were called and calculated with the matching Mutect2-based procedure for non-trio BD cases (N = 7720, excluding 660 samples without consent for reanalysis, median mitochondrial depth of 786×). We compiled the pHets in Wei et al. [55], which reported mitochondrial heteroplasmic variants using WGS data from 1526 duos (mother and offspring, mean mitochondrial depth of the offspring = 1901×) with a different pipeline from ours by selecting de novo heteroplasmic variants in the original publication. The Wei et al. study analyzed the samples from NIHR BioResource (Rare Diseases and 100,000 Genomes Project Pilot studies), including the patients affected by rare diseases and their relatives across several different disease domains (see ref. [55] for details). While the pHets in ToMMo GenII were called by the matching procedure for BD trios, the pHets in Wei et al. study were called by a different pipeline. Since the pHets in Wei et al. study were not directly comparable to BD pHets, we regarded the pHets in Wei et al. study as referential datasets. The schizophrenia trio data (N of trios = 615) were derived from Fromer et al. [50] (dbGaP phs000687.v1.p1) with authorization and processed by the matching procedure for the BD trios. The mitochondrial variants from other data sources underwent the same position-based filtering as BD. We limit the statistical analysis to high-confident SNVs with an MQ of 60.

Statistical Analysis

We performed the above statistical analysis with R-3.4.2 unless otherwise specified (DNENRICH and STRING). We used the following packages in R: pwr and lsa. The statistical assessments for rare proportions were performed by Fisher's exact test (FET). All the software packages used for the analyses are publicly available.

Data availability

The conventional WES data from study participants who provided informed consent for database registration are available through the National Bioscience Database Center (NBDC) Human Database, Japan, with accession code JGAS000273/JGAD000379. The DES data can only be accessed via formal collaboration. We used the following data from

the public database: WES from trios with schizophrenia (NCBI dbGaP phs000687.v1.p1 with authorization).

Supplementary Note

Methodological note on mosaic variant detection using peripheral tissues

Our study used peripheral tissues to detect mosaic variants of early developmental origin. While the mosaic variants in the brain are the direct target for psychiatric research, mosaic variants of early developmental origin should be basically shared among various tissues, including the brain and peripheral tissues. As we discussed previously [14], mosaic variants arising during the first five cell divisions (postzygotic cleavages before the differentiation of ectoderm, mesoderm, and endoderm) have VAFs of 1.56% (1/64) or more in one tissue when assuming symmetrical cell divisions in the development. The expected rate of such mosaic variants in 300× DES is 1.0 per sample. Since some mosaic variants have VAFs of less than 1.56% due to asymmetrical cell divisions, we set 1% as the threshold for mosaic variants of early developmental origin. Indeed, Bae et al. reported that mosaic variants arising during the first five postzygotic cleavages typically had VAFs above 1% [25]. Bae et al. also reported that over 90% of the SNVs with variant allele fractions (VAFs) above 2% in at least one brain region had a nonzero VAF in another tissue [25]. This means peripheral tissues are useful surrogates targeting mosaic variants with VAF ≥ 2% in the brain, complementing mosaic variant detection using brain samples. In practice, several studies have reported the contribution of mosaic variants to ASD using peripheral tissues [18-21]. While brain-specific mosaic variants arising later in development could also contribute to psychiatric disorders, the characteristics of brain mosaic variants in BD are beyond our scope. Mosaic variants in neurons were reported to have the following characteristics: non-clonal expansion, enrichment in coding exons and the genes involved in neural function, and transcriptional strand bias [84,85]. The association of brain-specific mosaic variants and BD should be addressed in future investigations.

Mosaic variants in the blood include mosaic variants of early developmental origin, but we cannot exclude possible contamination of mosaic variants specific to blood cells resulting from clonal expansion of hematopoietic cells. Two studies [60,61] reported that mosaic variants related to clonal hematopoiesis (CH-related mosaic variants) are rare in the samples derived from individuals under the age of 40 (e.g., frequency < 0.1% with whole exome sequencing [61]). Watson et al. calculated the relationship between ages and the frequency of CH-related mosaic variants with regard to the VAF detection thresholds, summarizing nine reports on the landscape of CH-related mosaic variants [62]. According to their calculation, CH-related variants of VAF > 2% are practically none

in individuals under 40, and CH-related variants of VAF > 0.3% are practically none in individuals under 30. Although they did not show direct data about CH-related variants of VAF > 1% (the threshold of our study), the frequency of CH-related variants of VAF > 1% is below 0.05 in individuals under 40. Thus, mosaic variants detected from the participants in their 10s or 20s are less likely to be CH-related, while mosaic variants detected from the participants in their 40s or 50s might include CH-related ones with a certain frequency (e.g., around 0.05 in individuals at the age of early 40s). Although the possible contamination of CH-related mosaic variants is problematic, CH-related mosaic variants can be excluded by assaying other peripheral tissues, e.g., nails and hairs, as tissues sharing the ectodermal developmental origin with the brain. Our study assayed nail samples from limited individuals with mosaic variants potentially relevant to BD in this study. Future investigations will benefit from a systematic collection of peripheral tissues of ectodermal origin.

We also analyzed DES data from 39 controls to check if the enrichment of DD/ASD genes in the mosaic variants in BD (our hypothesis) was primarily derived from the clonal expansion of hematopoietic cells. We observed no deleterious mosaic variants in DD genes and one deleterious mosaic variant in ASD genes (p.Trp1981* in *LRP1*) in 39 controls of our recruitment. The contrast of mosaic variant rates of 9/231 (0.039) in BD vs. 0/39 (0.00) in controls for DD genes and 14/231 (0.061) in BD vs. 1/39 (0.026) in controls for ASD genes did not contradict our hypothesis. While this contrast preliminarily supported our hypothesis, the statistical power was insufficient to assess the enrichment due to a small sample size of controls (for reference, $P = 0.366$ and 0.704 for DD and ASD genes, respectively, by two-sided FET). A larger sample size of controls will be required for a definite conclusion.

Despite the surrogate nature of peripheral tissues, using peripheral tissues has two advantages for mosaic variant investigation for psychiatric disorders. First, the peripheral tissues can be used for clinical genetic diagnosis. While postmortem brain samples are the best materials for neuroscientific research, brain specimens are usually unavailable from a living patient. In contrast, peripheral tissues have clinical utility for genetic diagnosis and risk prediction. Second, the high accessibility of peripheral tissues enables feasible expansion of sample size and disease categories. Postmortem brain samples are limited and relatively difficult to obtain. Postmortem brain samples for some diseases are rare. Peripheral tissues can complement this limitation of brain samples.

From the reasons and rationales above, using peripheral tissues is practical to detect mosaic variants potentially related to psychiatric disorders. Future studies using blood/saliva tissues will benefit from recruiting young participants (e.g., ages at

recruitment < 30) to prevent the possible contamination of CH-related mosaic variants, as well as sampling other peripheral tissues such as nails and hair.

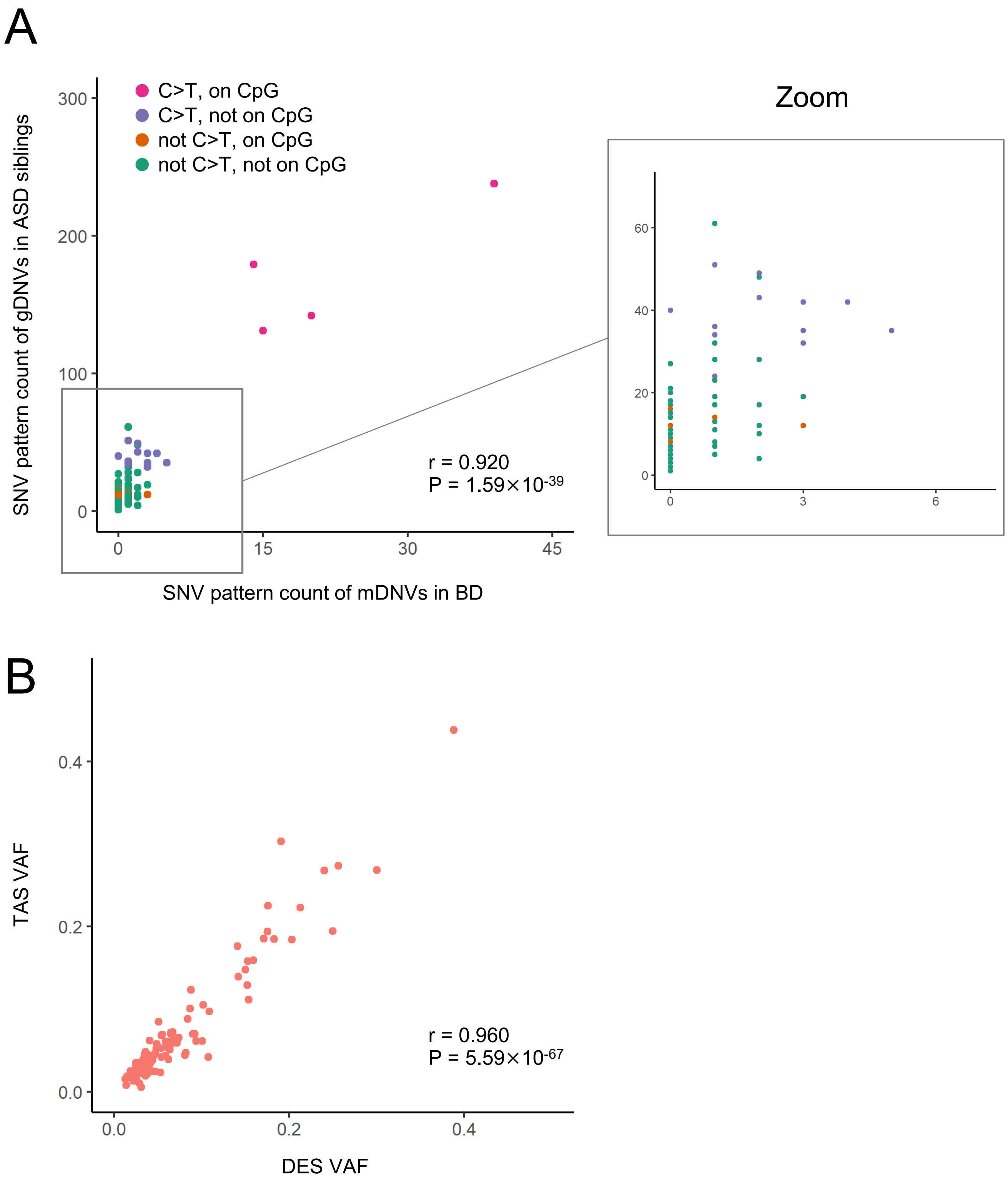

Figure S1. The mutation patterns and VAFs of mDNVs in BD

A) The correlation of 96 SNV pattern counts with trinucleotide contexts (e.g., ACG>ATG) between mDNVs in BD and referential gDNVs in unaffected ASD siblings. The Pearson correlation coefficient and its P-value (two-sided) are described on the right lower side. The Zoom view indicates the SNV patterns of small counts. The cosine similarity of SNV pattern counts between mDNVs in BD and gDNVs in ASD siblings is 0.901. **B)** The correlation of VAFs in DES (deep exome sequencing) and TAS (target amplicon sequencing). The correlation coefficient and its P-value are described on the right lower side.

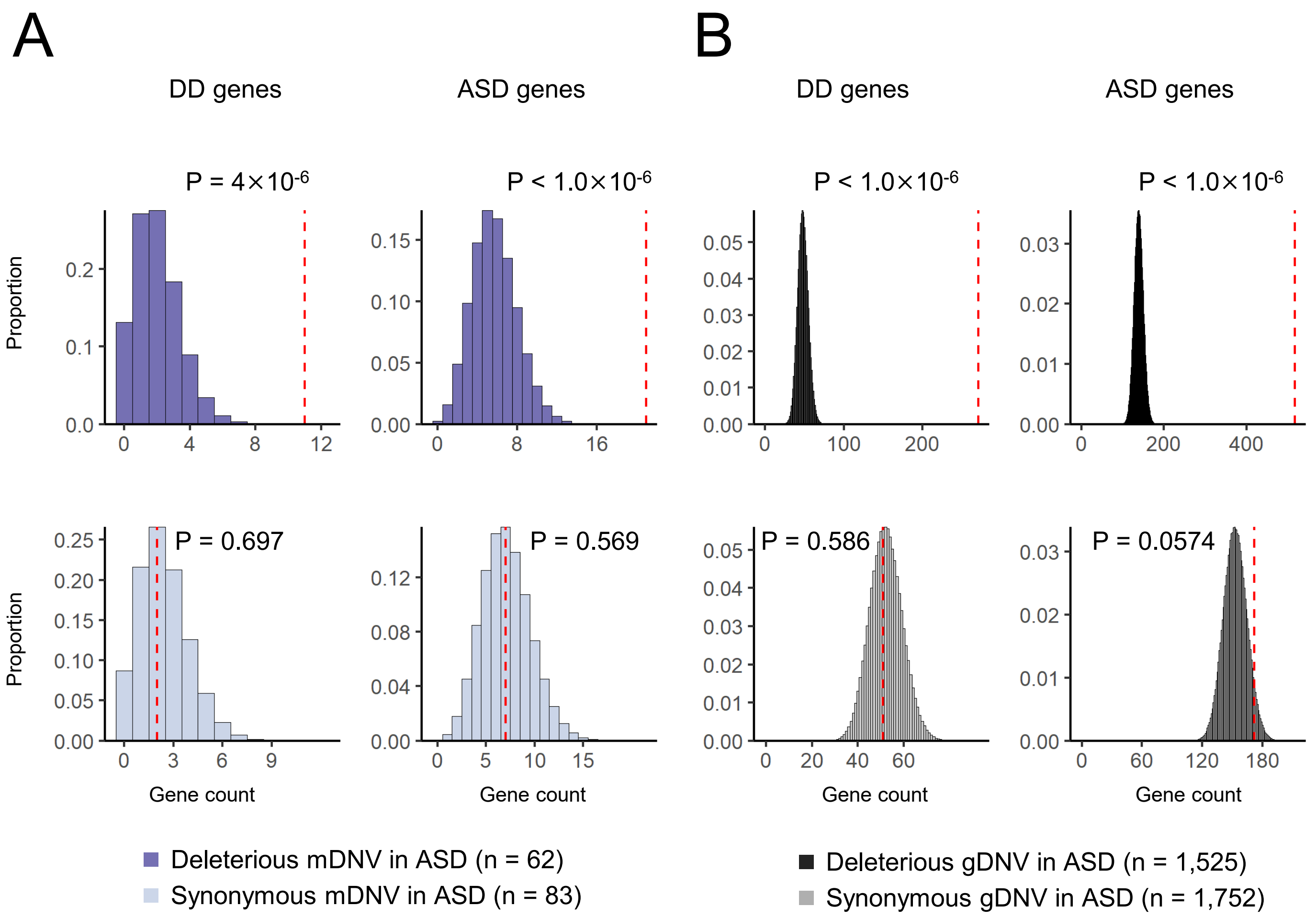

Figure S2. The enrichment of DD/ASD genes in the genes hit by mDNVs/gDNVs in ASD
The histogram illustrates the expected number of mDNVs/gDNVs hitting the two gene sets (DD and ASD genes, the x-axis) and their relative frequency (the y-axis). DNENRCIH calculated the distribution with one million random permutations. The red dotted line indicates the observed number of variants in DD/ASD genes. The probability of the observed number or more in the simulated distribution is described near the red dotted line. **A)** deleterious and synonymous mDNVs in ASD. **B)** deleterious and synonymous gDNVs in ASD.

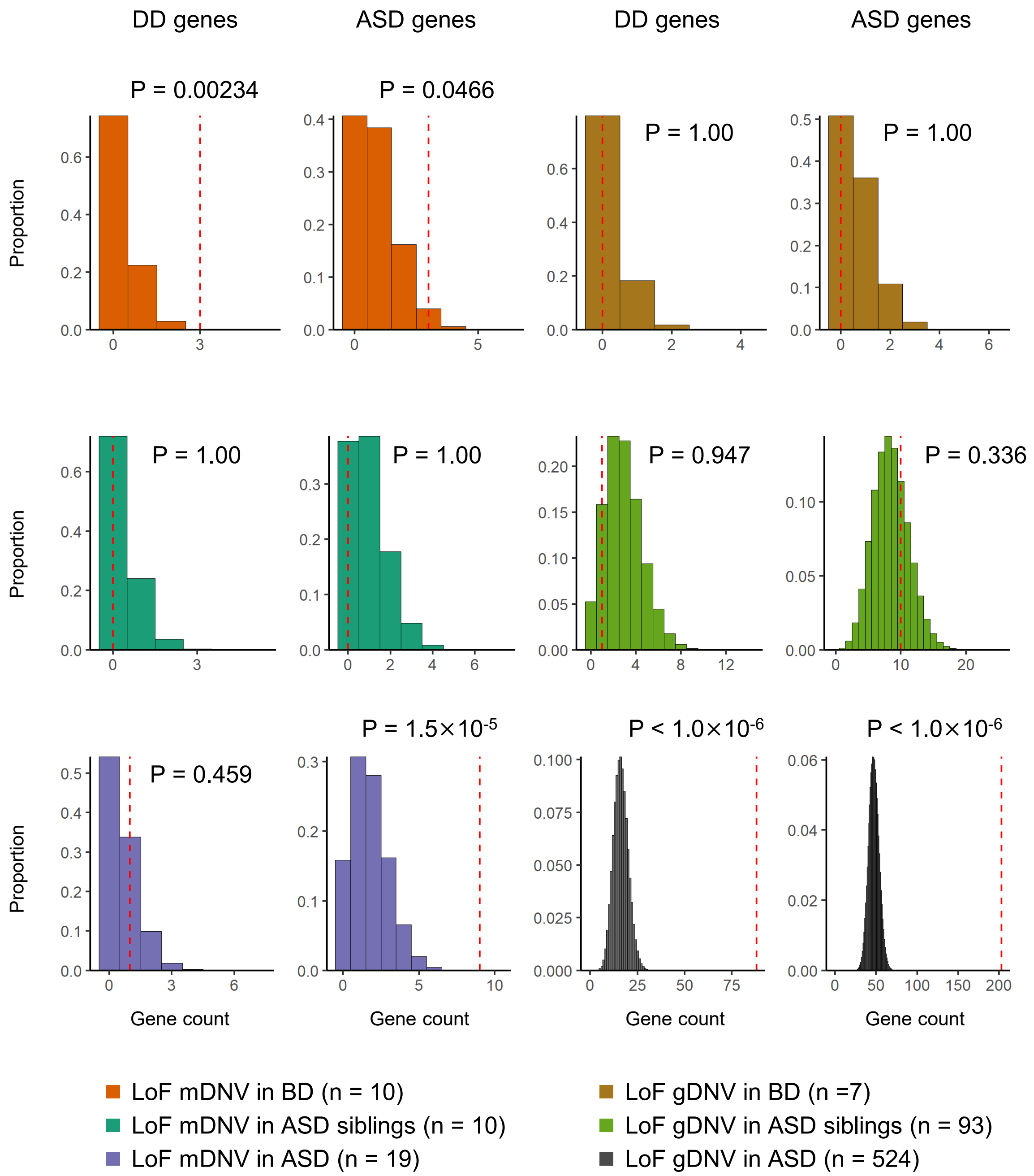

Figure S3. DNENRICH analysis of LoF variants

The histogram illustrates the expected number of LoF variants hitting the two gene sets (DD and ASD genes, the x-axis) and their relative frequency (the y-axis). DNENRCIH calculated the distribution with one million random permutations. The red dotted line indicates the observed number of variants in DD/ASD genes. The probability of the observed number or more in the simulated distribution is described near the red dotted line. The LoF variants in the mDNVs/gDNVs in BD/ASD siblings/ASD are indicated by different colors denoted on the lower side.

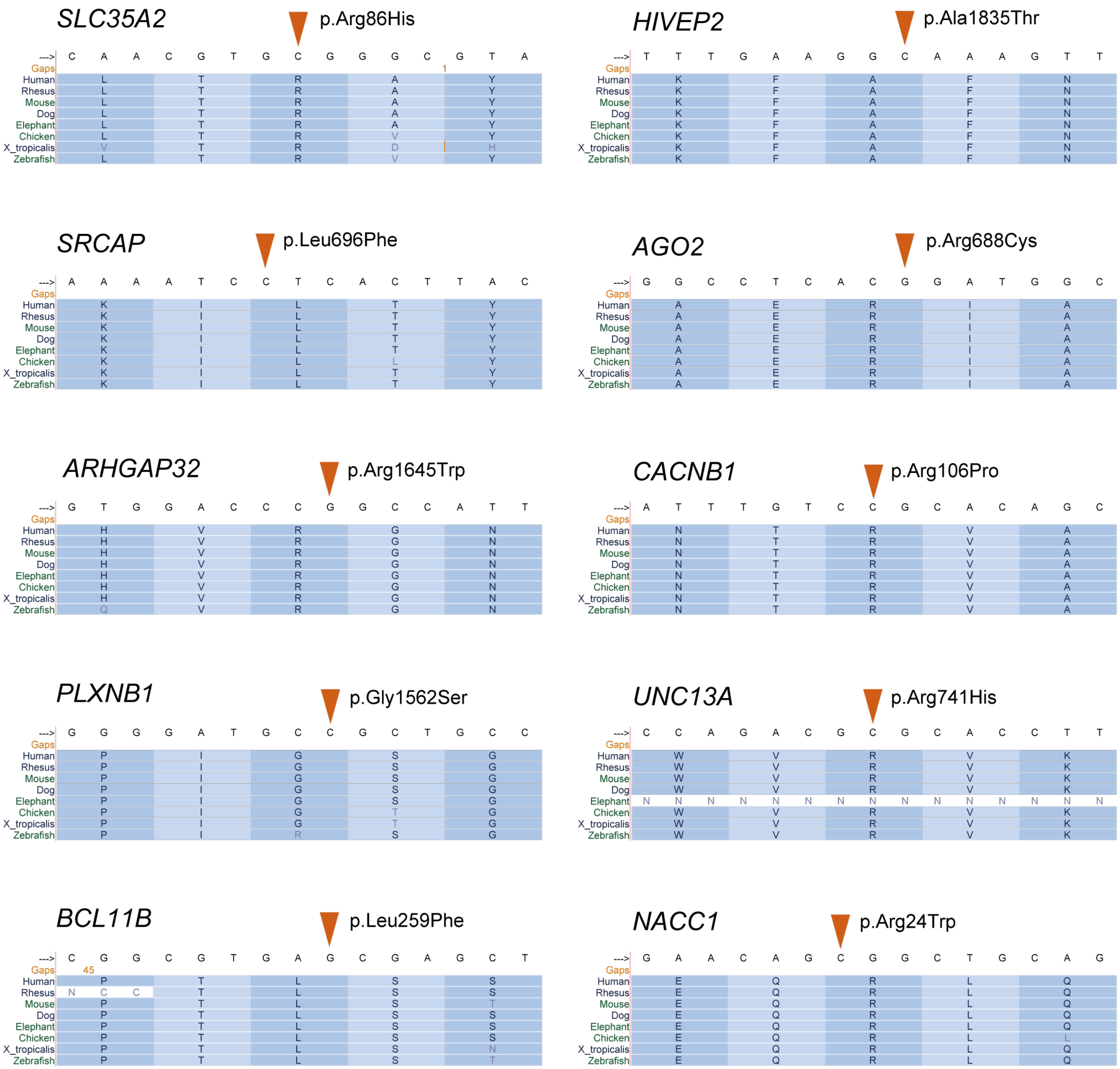

Figure S4. The amino acid conservation around the damaging missense mDNVs

The evolutionary conservation of the amino acid sequences around the damaging missense mDNVs in DD/ASD genes in BD. The amino acid sequences are derived from the Multiz Alignments of 100 Vertebrates of the UCSC Genome Browser (http://genome.ucsc.edu/). The amino acid sequences around the mDNVs (orange triangles) are highly conserved across the vertebrates.

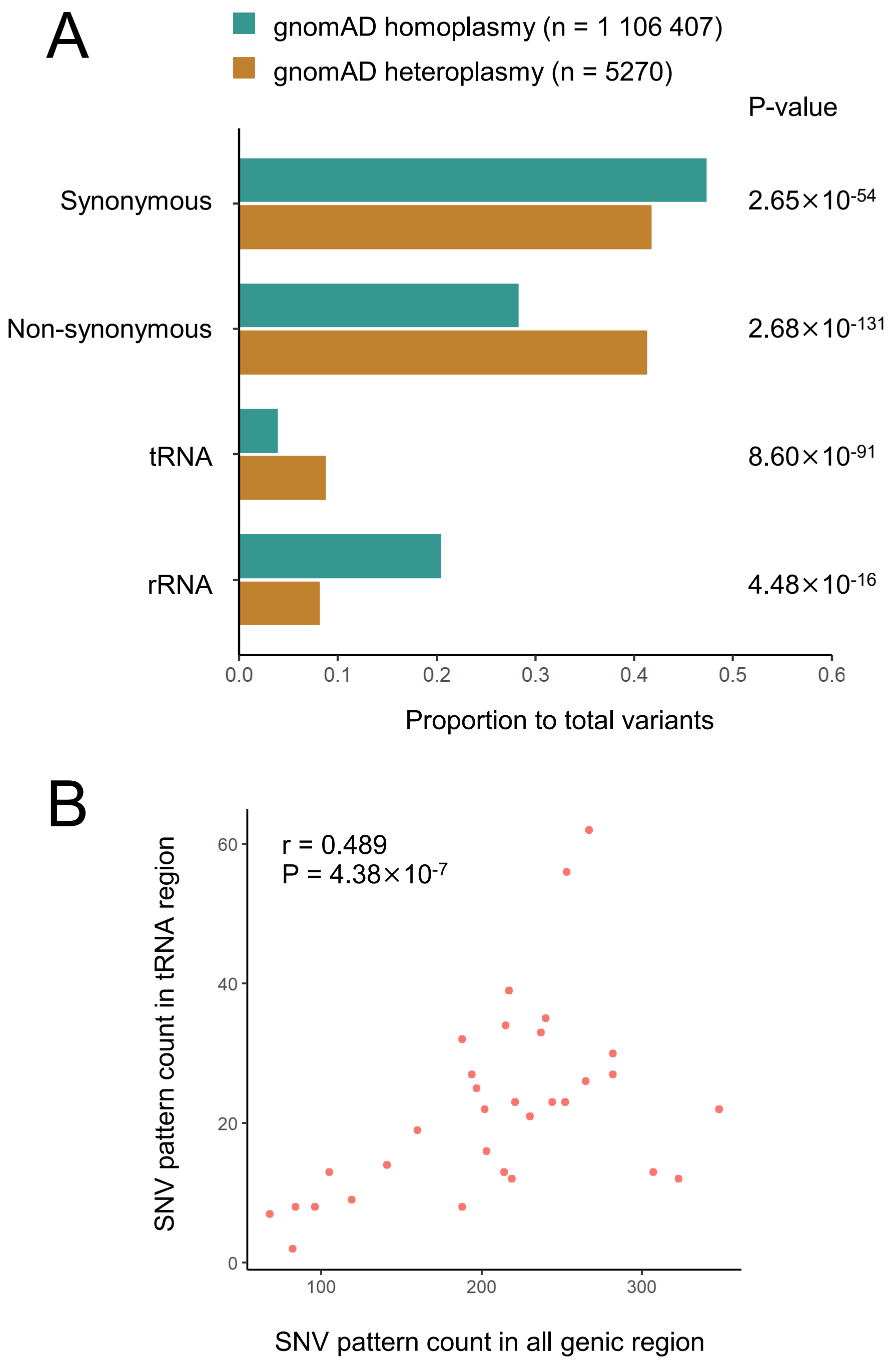

Figure S5. The mitochondrial variant analysis related to Figure 4

A) The comparison of homoplasmic and heteroplasmic variants in gnomAD-v3.1 (N = 56 383 individuals). The mitochondrial variants were limited to genic variants after the matching Panel of Normals (PoN) and low-mappability filters applied to BD datasets as a reference. The mitochondrial genic variants are classified as synonymous, non-synonymous, tRNA, rRNA, and intergenic. The x-axis indicates the proportions of each mitochondrial variant class to the total genic variants. The P-values calculated by two-sided Fisher's exact tests are described on the right side. **B)** The correlation of 96 SNV pattern counts with trinucleotide contexts (e.g., ACG>ATG) in tRNA and all genic variants in the mitochondrial genome. The Pearson correlation coefficient and its P-value (two-sided) are described on the left upper side. The cosine similarity between the two SNV pattern counts is 0.894.

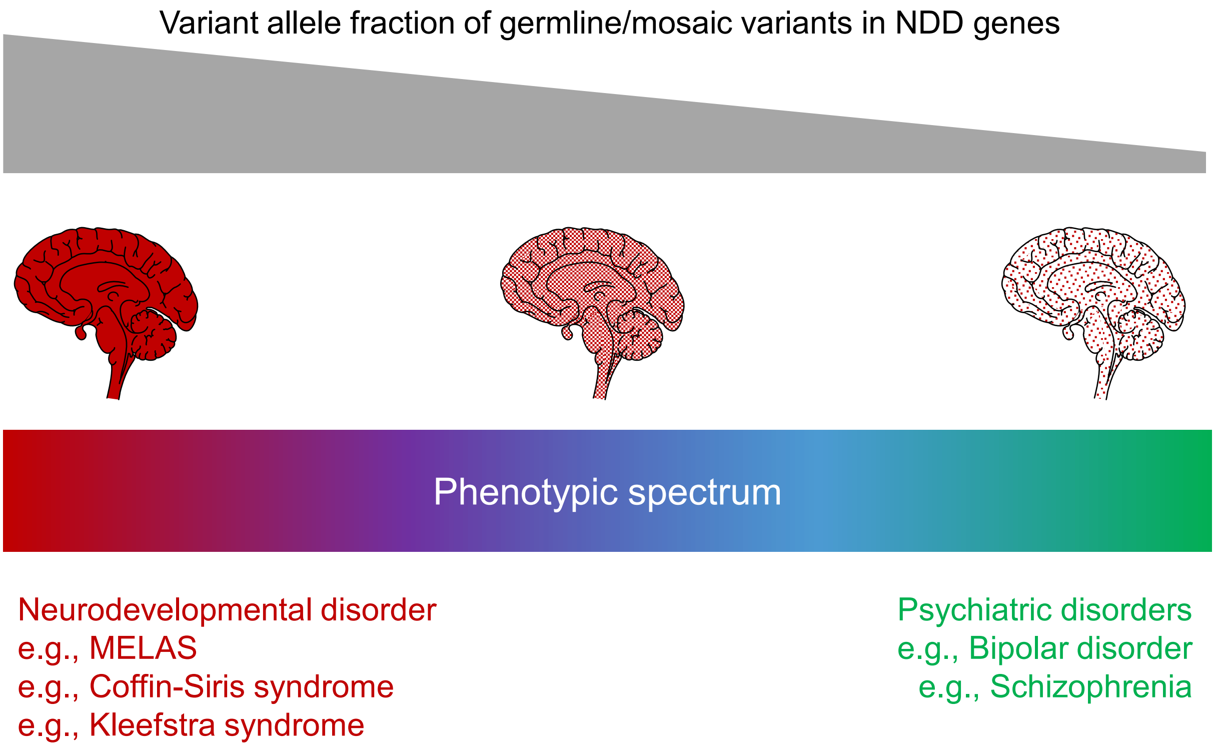

Figure S6. A model of phenotypic spectrum by mosaic variants
A schematic illustration of our model of the phenotypic spectrum by mosaic variants in neurodevelopmental disorder (NDD) genes.
